# NDUFS4, a mitochondrial complex I subunit, is essential for T-cell metabolic fitness and immune function

**DOI:** 10.3389/fimmu.2025.1734203

**Published:** 2026-01-07

**Authors:** Oded Shamriz, Zahala Bar-On, Omri Yosef, Leonor Cohen-Daniel, Ayelet Sheer, Or Reuven, Wajeeh Salaymeh, Amijai Saragovi, Raz Somech, Atar Lev, Hagar Mor-Shaked, Yuval Tal, Aviva Fattal-Valevski, Simon Edvardson, Michael Berger

**Affiliations:** 1 The Lautenberg Center for Immunology and Cancer Research, Institute of Medical Research Israel-Canada (IMRIC), Faculty of Medicine, The Hebrew University of Jerusalem, Jerusalem, Israel; 2 Allergy and Clinical Immunology Unit, Department of Medicine, Hadassah Medical Organization, Faculty of Medicine, The Hebrew University of Jerusalem, Jerusalem, Israel; 3 Department of Biochemistry, University of Washington, Seattle, WA, United States; 4 Institute for Protein Design, University of Washington, Seattle, WA, United States; 5 Pediatric Department A and Immunology Service, Jeffrey Modell Foundation Center, Edmond and Lily Safra Children’s Hospital, Sheba Medical Center, Tel Hashomer, Sackler Faculty of Medicine, Tel Aviv University, Ramat-Gan, Israel; 6 Sackler Faculty of Medicine, Tel Aviv University, Tel Aviv, Israel; 7 Department of Genetics, Hadassah Medical Center, The Faculty of Medicine, Hebrew University of Jerusalem, Jerusalem, Israel; 8 Pediatric Neurology Institute, Tel-Aviv Sourasky Medical Center, Tel-Aviv, Israel; 9 Pediatric Neurology Unit, Hadassah Medical Organization, Faculty of Medicine, The Hebrew University of Jerusalem, Jerusalem, Israel

**Keywords:** leigh syndrome (LS), mitochondria, NDUFS4, NDUFS4 knockout mice, T cells

## Abstract

**Introduction:**

Mitochondrial metabolism is essential for T-cell function, but the roles of individual electron transport chain (ETC) components are unclear. Here, we aimed to explore the role of mitochondrial complex I (CI) subunit NADH:ubiquinone oxidoreductase iron-sulfur protein 4 (NDUFS4) in T-cell metabolic fitness and immunity.

**Methods:**

We used a T cell-specific Ndufs4 knockout mouse model to find that NDUFS4 deficiency disrupts CI function, leading to metabolic and redox imbalances. Additionally, T cells from a patient with Leigh syndrome induced by NDUFS4 loss-of-function were analyzed.

**Results:**

Ndufs4-deficient T cells exhibit impaired OXPHOS, reduced respiratory capacity, and increased glycolysis, accompanied by reactive oxygen species (ROS) accumulation and defective TCR-driven activation, including reduced proliferation and cytokine production. In vivo, Ndufs4(-/-) mice show T-cell lymphopenia and impaired humoral and cytotoxic immunity. Importantly, T cells from a single Leigh syndrome patient with an NDUFS4 loss-of-function variant showed similar defects, including impaired activation and proliferation.

**Discussion:**

These findings highlight the importance of NDUFS4 for human immunity and establish a mechanistic link between complex I dysfunction and T-cell immunodeficiency. Our results identify NDUFS4 as a key regulator connecting mitochondrial integrity to adaptive immune function.

## Introduction

Mitochondria exploit oxidizable substrates to transfer electrons from nicotinamide adenine dinucleotide (NADH) or flavin adenine dinucleotide (FADH2) to oxygen via a multistep redox reaction. This electron transport chain (ETC) is conveyed by a series of protein complexes embedded in the inner mitochondrial membrane and generates an electrochemical proton gradient across the inner mitochondrial membrane. This is used to produce ATP through a process known as oxidative phosphorylation (OXPHOS) and serves as the driving force for other mitochondria-related physiological processes (1).

Complex I (CI), the first enzymatic complex of the ETC, oxidizes NADH and passes the electrons to ubiquinone via a series of protein-coupled redox centers (2). One of the 45 CI subunits, NADH:ubiquinone oxidoreductase iron-sulfur protein 4 (NDUFS4), is a nuclear-encoded protein that provides structural stability to the entire complex (3). NDUFS4 deficiency in humans and murine models, is known to cause Leigh syndrome, a progressive disorder characterized by neurological regression with motor and neuroradiological evidence of basal ganglia involvement (4).

T cells are the primary adaptive immune cells and have developed a versatile metabolic network to cope with the diverse metabolic environments encountered during inflammation. The metabolic reprogramming in activated and effector T cells plays a crucial role in regulating their function, determining their fate and regulating the overall immune function (5, 6).

As the primary metabolic hub within cells, mitochondria play a central role in T-cell-mediated immunity. Ample evidence has demonstrated that changes in mitochondria-related metabolism and function significantly impact the fate of activated T cells (7–9). These reports are further supported by clinical observations of high rates of infection in patients with Leigh syndrome (10).

To better understand the role of NDUFS4 in T-cell biology we generated *Ndufs4*^(-/-)^ mice with a T-cell-specific knockout. These mice exhibited T-cell lymphopenia, reduced homeostatic expansion, and a shift in T-cell subsets towards memory-like and effector-like phenotypes. Functionally, *Ndufs4*^(-/-)^ mice demonstrated impaired CD4^+^ T-cell-mediated humoral responses and reduced CD8^+^ T-cell-mediated immunity. Metabolic analyses revealed that *Ndufs4*^(-/-)^ T cells had compromised OXPHOS due to defective CI assembly, leading to a metabolic shift towards aerobic glycolysis and increased production of reactive oxygen species (ROS). Lastly, we analyzed T-cell effector functions in a patient with Leigh syndrome and recurrent infections due to an *NDUFS4* loss-of-function (LOF) gene variant. In line with our hypothesis, we found that the patient had impaired T-cell activation and proliferation. Our results emphasize the significance of NDUFS4 in T-cell mediated-immunity and metabolism.

## Results

### NDUFS4 knockout leads to T-cell lymphopenia due to impaired homeostatic expansion

To examine the role of NDUFS4 in T-cell immunity, we generated a T-cell-specific *Ndufs4*^(-/-)^ mouse model by crossbreeding *Ndufs4^(loxp/loxp)^* with *distal (d)LCK-CRE* transgenic mice. The resulting mice, referred to as *Ndufs4*^(-/-)^, had an excision of exon 2 of *Ndufs4* specifically in the T cell lineage (**Supplementary Figure S1A**). To confirm knockout in *Ndufs4*^(-/-)^ T cells, we used PCR to amplify a 388 bp segment that is expected to be generated following the excision of exon 2 by the *dLCK-CRE* recombinase in T lymphocytes (**Supplementary Figure S1B**). Immunoblot analysis using an anti-Ndufs4 antibody confirmed the absence of Ndufs4 protein expression in *Ndufs4*^(-/-)^ T cells (**Supplementary Figure S1C**). The representative PCRs for *Ndufs4 ^(loxp/loxp)^* and *LCK-CRE* transgenic mice are shown in **Supplementary Figures S1D, E**.

**Figure 1:**
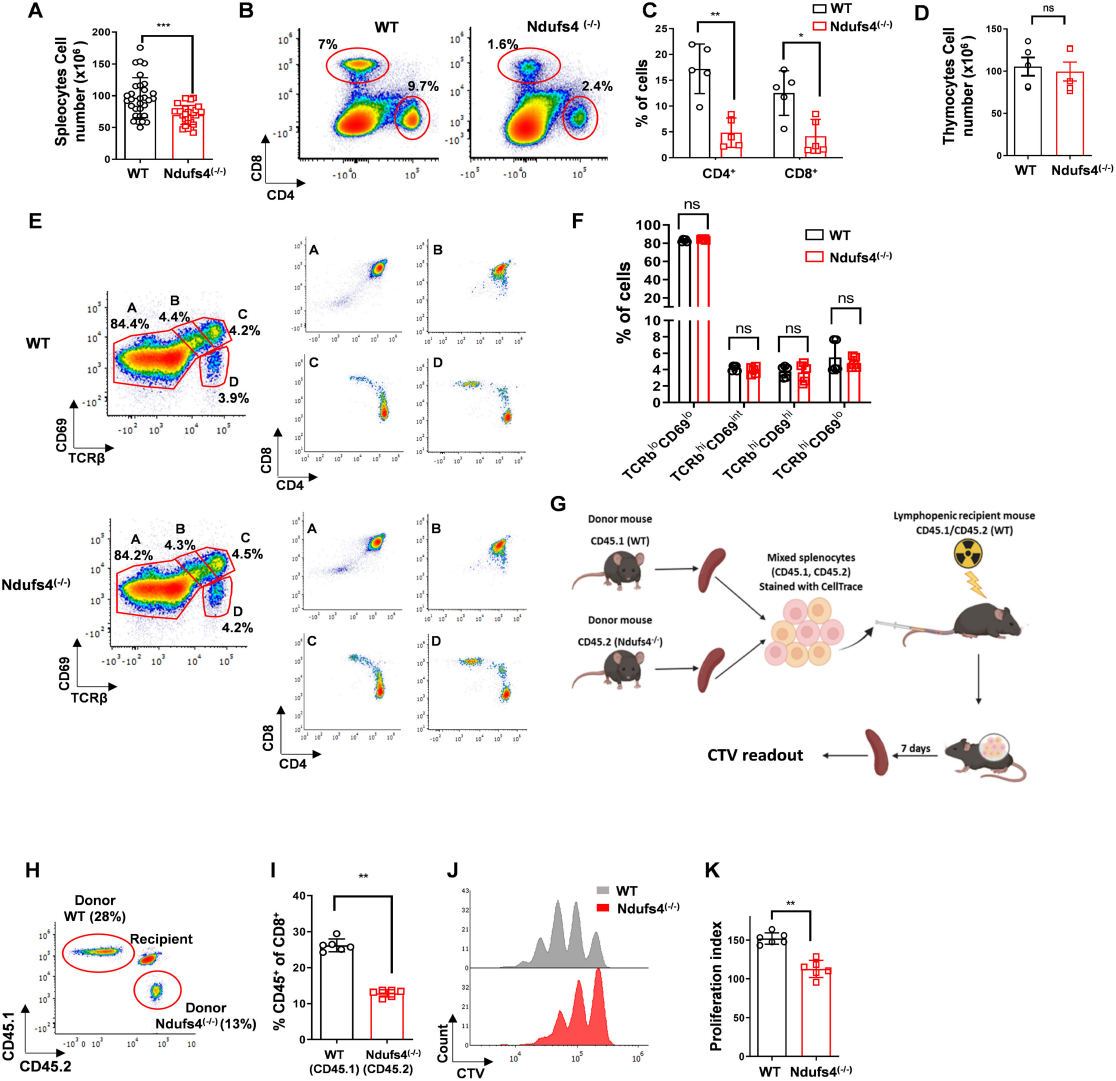
*Ndufs4*^(-/-)^ mice are characterized by lymphopenia due to impaired homeostatic expansion. **(A)** Absolute cell counts in the spleens of *Ndufs4*^(-/-)^ and WT mice (n=31 mice per group). **(B)** Representative flow cytometry plots illustrating the frequency of CD4^+^ and CD8^+^ T cells in the spleen of WT and *Ndufs4*^(-/-)^ mice. **(C)** Bar graph summarizing the data from **(B)**, showing the percentage of CD4^+^ and CD8^+^ T cells (n=5 mice per group). **(D)** Absolute thymocytes counts in of *Ndufs4*^(-/-)^ and WT mice (n=5 mice, each). **(E, F)** Assessment of thymic development in WT and *Ndufs4*^(-/-)^ mice at 5 weeks old. (E, left panel) Flow cytometry plots depicting the four developmental stages **(A-D)** based on CD69 versus TCRβ staining of whole thymocytes. (E, right panel) CD4 versus CD8 staining within each developmental stage **(A-D)**. **(F)** Bar graph summarizing the data from (E, left panel) (n=5 mice per group). **(G)** Diagram of the *in-vivo* adoptive T-cell transfer model used to study homeostatic expansion. Recipient CD45.1/CD45.2 heterozygous mice were sub-lethally irradiated to induce lymphopenia. Donor WT (CD45.1^+^) and *Ndufs4*^(-/-)^ (CD45.2^+^) mice were sacrificed, and splenocytes were isolated, mixed at an approximately 1:1 ratio, and labeled with CTV. Transferred cell numbers were normalized to total splenocyte count. The CTV-labeled splenocyte mixture was then intraperitoneally injected into the recipient lymphopenic mice. **(H)** Representative flow cytometry plots showing CD45.1 versus CD45.2 staining of splenocytes from recipient mice, 7 days post-transfer. Cells are gated on CD8^+^ T cells **(I)** Bar graph summarizing the data from **(H, J)** Representative stacked histogram of CTV intensity in donor CD8+ T cells. **(K)** Bar graph summarizing the proliferation index of donor CD8^+^ T cells from **(J)** (n=6 mice per group). Statistical analysis was performed using the two-tailed unpaired Mann–Whitney test. Data are presented as mean values ± SD (P values: *P ≤ 0.05, **P ≤ 0.01, ***P ≤ 0.001).

Next, to explore the immunological effects of Ndufs4 deficiency, we conducted immune phenotyping using flow cytometry. Absolute splenocyte counts, as well as splenic CD4^+^ and CD8^+^ percentages, were reduced in *Ndufs4*^(-/-)^ compared to wild-type (WT) mice (**Figures 1A-C**). T-cell lymphopenia was also observed in the peripheral blood (**Supplementary Figure S2A**) and cervical lymph nodes (LNs) (**Supplementary Figures S2B**-**D**) of *Ndufs4*^(-/-)^ mice. However, absolute thymocyte counts were comparable between *Ndufs4*^(-/-)^ and WT mice (**Figure 1D***).* Importantly, lymphopenia was not observed in CD19^+^ B cells from *Ndufs4*^(-/-)^ mice, demonstrating that the dLCK-Cre: *Ndufs4^(loxp/loxp)^* model exhibits a selective defect in T cells (**Supplementary Figure S2A***)*.

Analysis of T-cell subsets by staining for the T-cell maturation markers CD44, CD62L, and CD122 revealed significant changes in *Ndufs4*^(-/-)^ mice. Specifically, there was a decrease in naïve T cells, which are characterized as CD44^lo^ CD62L^hi^. Alternatively, there was a rise in memory-like T cells, characterized by CD44^hi^ CD62L^hi^ and CD122^+^, along with a rise in effector-like T cells, characterized by CD44^hi^ CD62L^lo^ (**Supplementary Figures S2E**-**I**). These findings are consistent with previous studies showing that increased memory-phenotype T cells are a well-characterized hallmark of T-cell lymphopenia in mice. This shift is driven by lymphopenia-induced homeostatic proliferation, which generates CD44^hi^ CD122^hi^ memory-like cells (11, 12).

Next, we aimed to understand the underlying reason for the T-cell lymphopenia in *Ndufs4*^(-/-)^ mice. We began by analyzing thymic development through flow cytometry. The proportions of double-positive (CD4^+^CD8^+^) and single-positive (CD4^+^CD8^-^ or CD8^+^CD4^-^) cell populations were similar between WT and *Ndufs4*^(-/-)^ mice (**Supplementary Figures S3A, B**). Moreover, the distribution and phenotype of pre-positive selection thymic subsets, CD69^-^TCRβ^-^ and CD69^int^TCRβ^int^, as well as post-positive selection thymocytes, CD69^+^TCRβ^hi^ and CD69^-^TCRβ^hi^, were similar between the two groups (**Figures 1E, F**). These findings indicate that thymic development is unaffected in *Ndufs4*^(-/-)^ mice, suggesting that other factors may be responsible for the observed T-cell lymphopenia.

Next, we tested the homeostatic expansion capacity of naïve *Ndufs4*^(-/-)^ T cells in the secondary lymphatic system as the cause for the observed T-cell lymphopenia in Ndufs4^(-/-)^ mice. For this, we adoptively transferred splenocytes into lymphopenic mice, thus analyzing naïve T cells (T_N_) proliferation *in-vivo* (**Figure 1G**). Sub-lethally irradiated recipient WT mice heterozygous for CD45.1/CD45.2 were adoptively transferred with donor CTV-labeled splenocytes derived from CD45.1 WT and CD45.2 *Ndufs4*^(-/-)^ mice, containing T cells at an approximately 1:1 ratio. Seven days post-transfer, the spleens of the recipient mice were harvested. Naïve T cells from WT and *Ndufs4*^(-/-)^ donor mice and the signal of CellTrace were then quantified using flow cytometry, thus assessing T_N_ homeostatic expansion. The analysis showed a reduced count of donor *Ndufs4*^(-/-)^ T_N_ cells in comparison to WT T_N_ cells (**Figures 1H, I**; **Supplementary Figures S3C, D**, for CD8^+^ and CD4^+^ T cells, respectively; *P ≤ 0.01*). Additionally, *Ndufs4*^(-/-)^ CD8^+^ (**Figures 1J, K**) and CD4^+^ (**Supplementary Figures S3E, F**) T cells demonstrated significantly lower proliferation rates than the WT T cells. Finally, we investigated whether T-cell lymphopenia in *Ndufs4*^(-/-)^ mice was due to increased apoptosis. To this end, CD8^+^ T-cell splenocytes were stained with propidium iodide (PI) and Annexin V. Cells were analyzed across four subpopulations: necrotic (PI^+^Annexin V^-^), live (PI^+^;Annexin V^-^), early apoptotic (PI^+^Annexin V^-^) and late apoptotic (PI^+^Annexin V^-^). No significant differences were observed between WT and *Ndufs4*^(-/-)^ mice in any of these groups, indicating that T-cell lymphopenia in *Ndufs4*^(-/-)^ mice is not attributable to increased apoptosis (**Supplementary Figures S3G, H**).

Collectively, these data validate that *Ndufs4*^(-/-)^ mice are characterized by intact thymic development and T-cell lymphopenia, which probably resulted from impaired homeostatic expansion.

### Impaired T-cell–mediated immunity in *Ndufs4*^(-/-)^ mice

**Figure 2:**
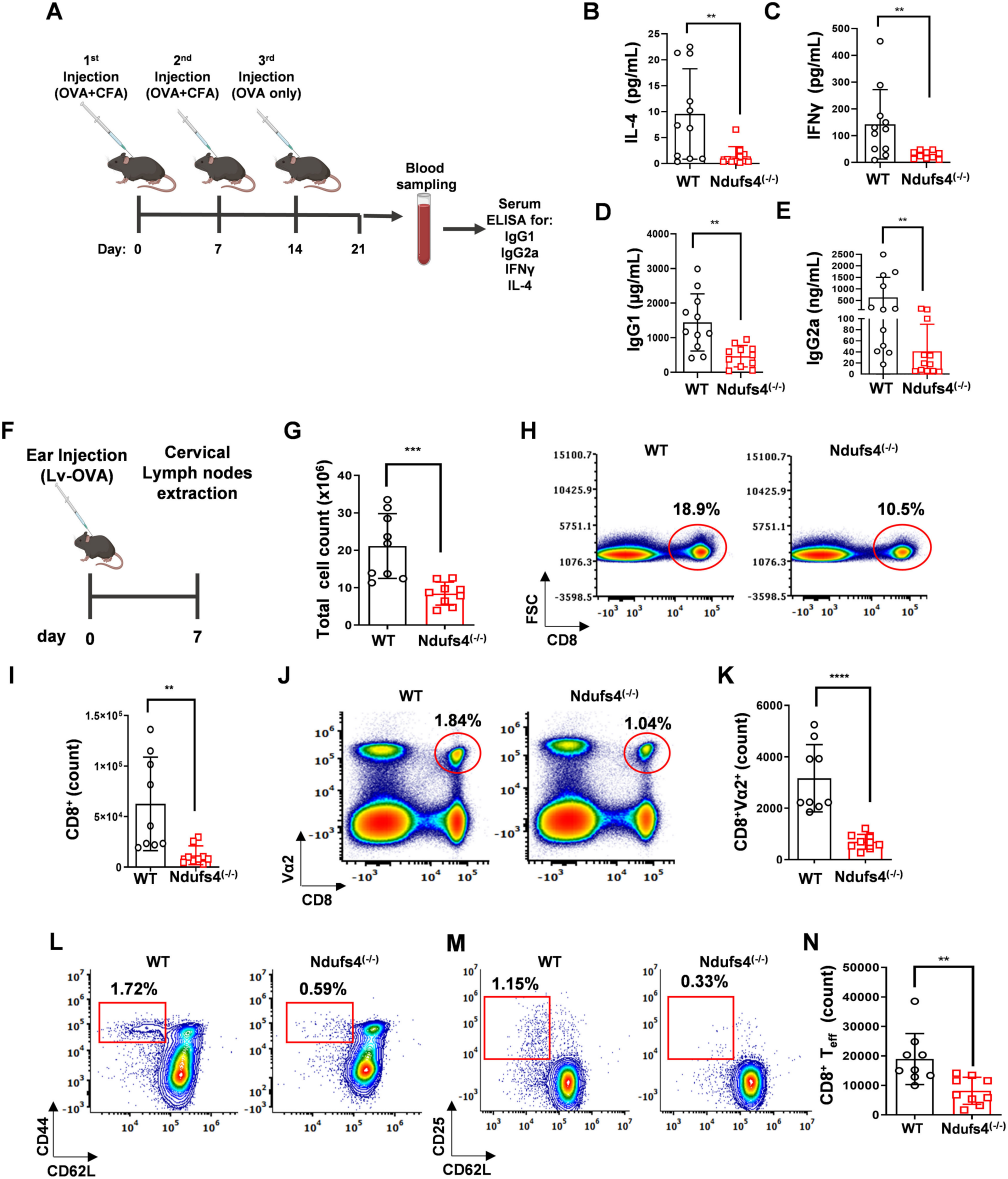
Impaired T-cell immune responses in *Ndufs4*^(-/-)^ mice. **(A)** Diagram of the *in-vivo* immunization model employed to investigate CD4^+^-mediated humoral responses. Ndufs4^(-/-)^ and WT mice were subcutaneously injected with ovalbumin (OVA) and complete Freund’s adjuvant weekly for two weeks, followed by a booster injection of OVA in the third week. One week after the booster, serum concentrations of the cytokines IL-4 **(B)** and IFNγ **(C)**, as well as OVA-specific IgG1 **(D)** and IgG2a **(E)**, were assessed using enzyme-linked immunosorbent assay (ELISA) (n=11 mice per group). **(F)** Diagram of the *in-vivo* infection model used to investigate CD8^+^ T cell activation. WT and *Ndufs4*^(-/-)^ littermate mice were intradermally primed in the left ear pinna with 5 × 10^6^ transduction units (TU) of a lentivirus expressing OVA (Lv-OVA). Seven days post-infection, cervical lymph nodes (LN) were harvested, and single cells were counted and analyzed for activation-related surface markers via flow cytometry. **(G)** Total cell counts from left cervical lymph nodes of WT and *Ndufs4*^(-/-)^ mice. **(H)** Representative flow cytometry plots showing CD8 versus FSC of cells from the left cervical lymph nodes of Lv-OVA-injected WT and *Ndufs4*^(-/-)^ mice. **(I)** Comparison of CD8+ T cell counts in the left cervical lymph nodes of WT and *Ndufs4*^(-/-)^ mice. **(J)** Representative flow cytometry plots of CD8 versus TCRVα2 (Vα2) in cells from the left cervical lymph nodes of Lv-OVA-injected WT and *Ndufs4*^(-/-)^ mice. **(K)** Bar graph summarizing the data from **(J)**, indicating the total number of CD8^+^Vα2^+^ cells. **(L, M)** Representative flow cytometry plots showing CD62L versus CD44 **(L)**, and CD62L versus CD25 **(M)**, gated on CD8^+^Vα2^+^ T cells. **(N)** Bar graph summarizing the results from **(L)** and **(M)** as the number of CD8^+^Vα2^+^CD44^+^CD25^+^CD62L^-^ cells in the cervical lymph nodes of WT and *Ndufs4*^(-/-)^ mice (n=9 mice per group). Statistical analysis was performed using the two-tailed unpaired Mann–Whitney test. Data are shown as mean values ± SD (P value, **P ≤ 0.01, *** p<0.001).

To assess CD4^+^ T-cell–mediated immunity, we immunized WT and *Ndufs4*^(-/-)^ mice with ovalbumin (OVA) in complete Freund’s adjuvant. We then measured Th1 and Th2 responses by quantifying OVA-specific IgG2a and IgG1 antibodies and the cytokines IFN-γ and IL-4 (**Figure 2A**). Vaccinated *Ndufs4*^(-/-)^ mice exhibited lower levels of IL-4 and IFN-γ in their sera compared to vaccinated WT mice (**Figures 2B, C**; *P ≤ 0.01*). Additionally, our findings indicated a significant decrease in both OVA-specific IgG1 and IgG2a antibodies in vaccinated *Ndufs4*^(-/-)^ mice relative to WT mice (**Figures 2D, E***; P ≤ 0.01*). These results indicate that the CD4^+^ T-cell-mediated humoral response is impaired in *Ndufs4*^(-/-)^ mice.

To analyze the activity of CD8^+^ T cells, we subsequently injected *Ndufs4*^(-/-)^ and WT mice intradermally in the left ear pinna with an OVA-expressing *lentivirus* (Lv-OVA) (**Figure 2F**). After 7 days, we assessed the phenotype of OVA-associated CD8^+^ T cells in the left cervical LNs by flow cytometry using the right LN as a control. Interestingly, the absolute cell counts in the left cervical LN of *Ndufs4*^(-/-)^ mice were significantly reduced compared to WT mice (**Figure 2G**). In addition, the absolute counts and percentages of total CD8^+^ and OVA-associated CD8^+^ (Vα2^+^) T cells in the left cervical LN were significantly reduced in the *Ndufs4*^(-/-)^ mice compared to the WT mice (**Figures 2H-K**). We quantified OVA-associated CD8^+^ T_eff_ cells expressing CD25^+^CD62L^lo^ or CD44^hi^ CD62L^lo^ and observed a reduction in both the percentages and absolute numbers of these cells in *Ndufs4*^(-/-)^ mice 1 week after Lv-OVA injection (**Figures 2L-N**). A comparison between the right and left cervical LNs validated that T-cell activation was specific to the injection site in the left ear pinna (**Supplementary Figures S4A**-**E**).

### Adoptive CD8^+^ T-cell transfer reveals impaired effector functions in *Ndufs4*^(−/−)^ mice

**Figure 3:**
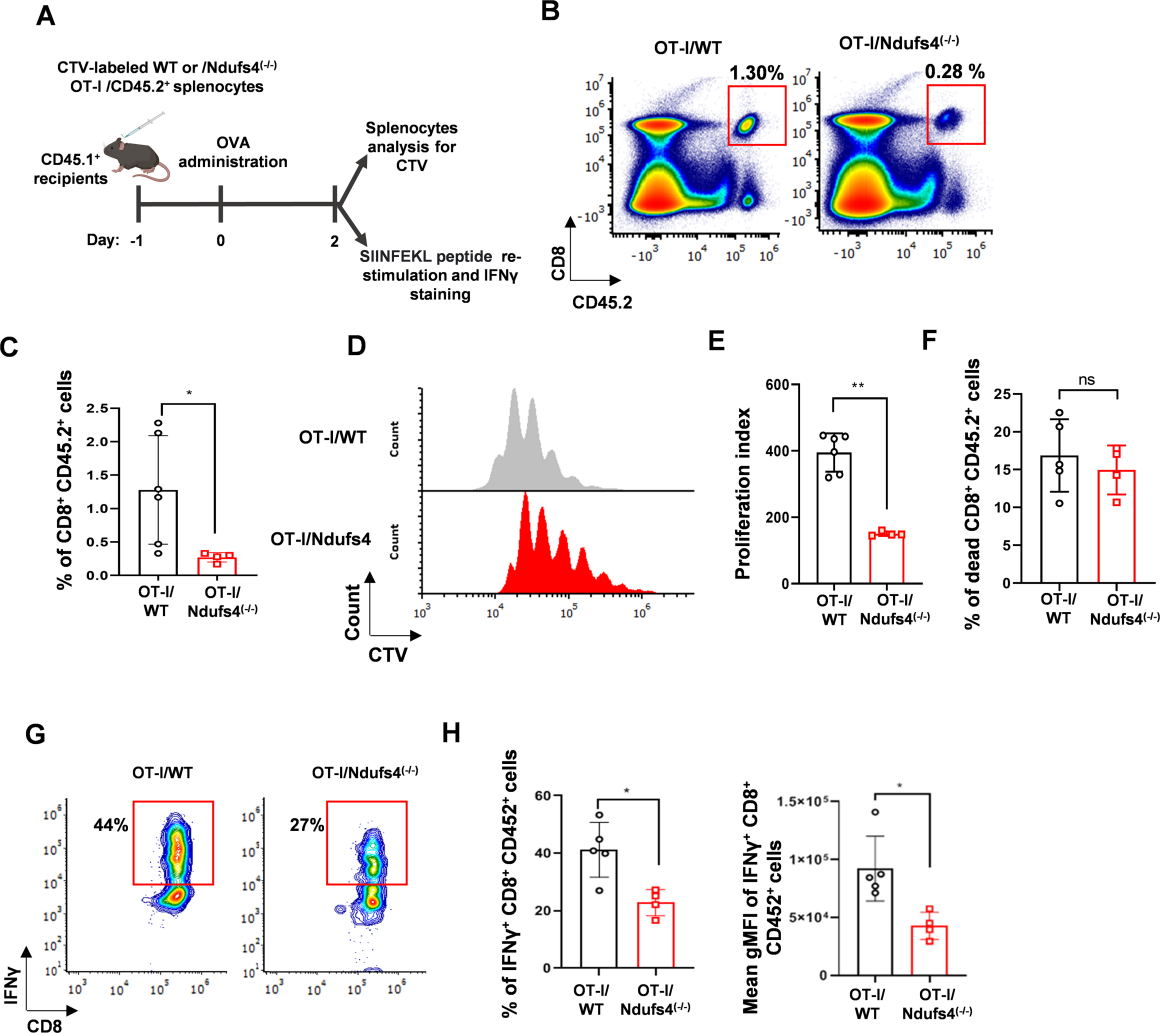
Adoptive T-cell transfer model used to assess effector function of *Ndufs4*^(-/-)^ CD8^+^ T cells. **(A)** Diagram of the adoptive T-cell transfer experiment. CTV-labeled donor T cells from OT-I/WT or OT-I/*Ndufs4*^(-/-)^ CD45.2^+^ mice were intravenously transferred into CD45.1^+^ WT recipients. The next day, OVA was administered intraperitoneally. Two days later, mice were sacrificed, spleens were collected, and CTV signal intensity was measured using flow cytometry. Additionally, splenocytes from treated mice were restimulated *in vitro* with the SIINFEKL peptide for 6 hours, followed by intracellular staining for IFNγ and flow cytometry analysis. **(B)** Representative flow cytometry plots of CD8 versus CD45.2 gated on total lymphocytes cells. The rectangular region marks the CD8, CD45.2 double-positive cells corresponding to the donor CD8^+^ T cells. **(C)** Bar graph summarizing the data as percentages of CD8^+^Vα2^+^ cells gated on CD45.2^+^ cells.” **(D)** CTV intensity of donor OT-I/WT or OT-I/*Ndufs4*^(-/-)^ CD8^+^ T cells. **(E)** Bar graph summarizing the data from **(F)** as the proliferation index. **(G)** Representative flow cytometry plots of CD8 versus IFNγ gated on CD8^+^CD45.2^+^ cells restimulated *in vitro* with SIINFEKL peptide. **(H)** Bar graph summarizing the data from **(G)** as percentages of IFNγ^+^ CD8^+^ CD45.2^+^ cells (n=5 mice per group). Statistical analysis was conducted using the two-tailed unpaired Mann–Whitney test. Results are shown as mean values ± SD (P values: ns- non-significant *P ≤ 0.05, **P ≤ 0.01).

To further analyze the activation of *Ndufs4*^(-/-)^ CD8^+^ T cells, we employed an *in-vivo* model of adoptive cell transfer (ACT) (**Figure 3A**). CTV-labeled OT-I/*Ndufs4*^(-/-)^ and OT-I/WT splenocytes were intravenously injected into CD45.1 recipient WT mice. The next day, both groups received an intraperitoneal injection of OVA. Two days after OVA administration, the mice were sacrificed, and the flow cytometry readout of CTV-labeled donor T cells was performed. In line with our previous findings, the ratio of donor OT-I*/Ndufs4*^(-/-)^ CD8^+^ T cells was reduced compared to their WT counterpart in recipient mice (**Figures 3B, C**). Consistent with these results, flow cytometry analysis of CTV confirmed that OT-I/Ndufs4^(-/-)^ CD8^+^ T cells exhibited reduced proliferation compared to OT-I/WT cells after 2 days of activation (**Figures 3D, E**; *P ≤ 0.01*), with no evidence of increased cell death (**Figure 3F**). Lastly, we tested the effector function of the donor T cells by re-stimulating them *ex-vivo* with the SIINFEKEL peptide for 5 hours and assessing IFN-γ production through intracellular staining. This analysis revealed diminished IFN-γ production by effector CD8^+^ T cells from OT-I/*Ndufs4*^(-/-)^ mice compared with their OT-I/WT counterparts (**Figures 3G, H**).

Overall, our results confirm impaired activation and function of *Ndufs4*^(-/-)^ CD8^+^ T cells.

### Complex I deficiency and reduced spare respiratory capacity in *Ndufs4*^(-/-)^ T cells

**Figure 4:**
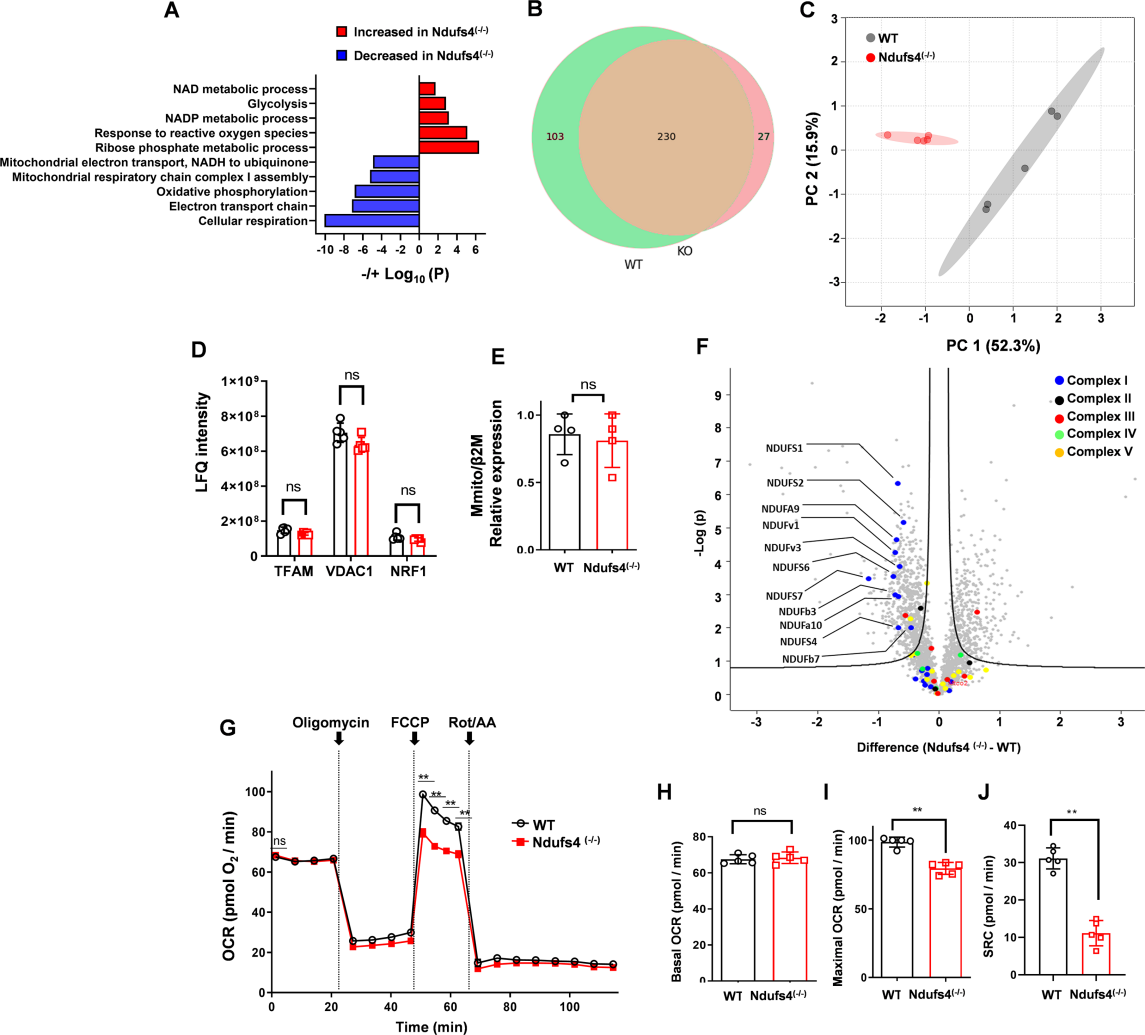
Mitochondrial complex I deficiency and decreased cellular respiration in *Ndufs4*^(-/-)^ CD8^+^ T cells. **(A-F)** Mass spectrometry (MS) analysis was performed on protein extracts from freshly isolated CD8^+^ T cells of *Ndufs4*^(-/-)^ and WT mice (n=5 mice per group). **(A)** Overview of pathway enrichment analysis showing proteins with increased (red bars) and decreased (blue bars) levels in *Ndufs4*^(-/-)^ compared to WT CD8^+^ T cells. **(B)** Venn diagram illustrating the overlap and differences in protein detection between naïve WT and *Ndufs4*^(-/-)^ CD8^+^ T cells. **(C)** Principal component analysis of log2-transformed data for mitochondrial-associated proteins, highlighting the first two principal components that capture the most variance. **(D)** Bar graph showing the levels of TFAM, VDAC1, and NRF1 as measured by Label-Free Quantitation (LFQ). **(E)** RT-PCR analysis of mitochondrial DNA (mtDNA) levels in freshly isolated CD8^+^ T cells from WT and *Ndufs4*^(-/-)^ mice, using primers for the mitochondrial gene RNR2, with normalization to the nuclear gene β2M. **(F)** Volcano plot depicting differentially expressed proteins based on relative peptide abundances, with the negative logarithm of p-values plotted against fold change. Mitochondrial complexes I-V-associated proteins are highlighted with color dots. **(G)** Dot plots of oxygen consumption rate (OCR) measured by Seahorse XF96 after sequential injections of Oligomycin [1 µM], FCCP [2 µM], and Rotenone + Antimycin-A [1 µM] (Rot+AA) in isolated naïve CD8+ T cells from WT (black) and *Ndufs4*^(-/-)^ (red) mice. **(H-J)** Bar graphs summarizing the OCR data shown in **(G)**, including basal OCR **(H)**, maximal OCR (after FCCP injection) **(I)**, and spare respiratory capacity (SRC) calculated as maximal OCR minus basal OCR **(J)**. Statistical analyses were conducted using Student’s t-test on log2-transformed data after Z-score normalization **(C, F)**, and two-tailed unpaired Mann–Whitney test **(D, E, G-J)**. Results are presented as mean values ± SD (P values: ns = non-significant; **P ≤ 0.01).

The mitochondria play a central role in T-cell activation and effector functions (7, 8, 13, 14). Considering its pivotal role in CI assembly, Ndufs4 deficiency is expected to lead to mitochondrial impairment and overall altered cellular metabolism, resulting in the observed T-cell defect in *Ndufs4*^(-/-)^. Therefore, we aimed to decipher the underlying mechanism inducing impaired T cells functionality by Ndufs4 deficiency. We used protein mass spectrometry (MS) to investigate changes in the cellular proteome. Comparative analysis of all altered proteins revealed a decrease in *Ndufs4*^(-/-)^ T cells of the proteins participating in cellular respiration, specifically in the ETC, and an increase in proteins that play a role in aerobic glycolysis and the response to redox/oxidative stress (**Figure 4A**). These results suggest that mitochondrial dysfunction is directly caused by the Ndufs4 deficiency, which forces metabolic adaptation to ETC impairment and disrupted OXPHOS. Therefore, we initially analyzed the influence of the *Ndufs4* deficiency on mitochondria. Comparing all of the detected mitochondrial proteins revealed that *Ndufs4*^(-/-)^ T cells have distinct mitochondria (**Figures 4B, C**). To evaluate alterations in the mitochondrial content, we examined the expression of known markers of mitobiogenesis. No significant alterations were found in the expression levels of voltage-dependent anion-selective channel 1 (VDAC1), nuclear respiratory factor 1 (NRF1), and transcription factor A, mitochondrial (TFAM) in *Ndufs4*^(-/-)^ T cells (**Figure 4D**). In addition, the mitochondrial DNA (mtDNA) content was comparable in *Ndufs4*^(-/-)^ and WT CD8^+^ T cells (**Figure 4E**). Finally, imaging mitochondria directly by confocal microscopy using MitoTracker far-red staining revealed comparable intensity, mitochondrial circularity, length, and area between Ndufs4^(-/-)^ and WT T cells (**Supplementary Figures S5A**-**E**). These results indicate that mitochondrial biogenesis is intact in *Ndufs4*^(-/-)^ T cells. Next, protein-set enrichment analysis indicated a decreased ETC, mainly reduced CI assembly and function in *Ndufs4*^(-/-)^ T cells (**Figure 4A**). Based on these findings, along with the known role of Ndufs4 in CI assembly and function, we focused our analysis on the proteome profile of the five mitochondrial complexes. Eleven of the 21 detected CI-specific subunits were decreased in *Ndufs4*^(-/-)^ T cells, whereas subunits belonging to other mitochondrial complexes were relatively preserved (**Figure 4F***; P ≤ 0.0001* for ndufv3, ndufs1, ndufsv1, ndufa9 and ndufs2; *P ≤ 0.001* for ndufs7, ndufb3, ndufs6 and ndufa10*; P ≤ 0.01* for ndufb7 and ndufs4). Lastly, we assessed how NDUFS4 deficiency impacts mitochondrial function in T cells. Using Seahorse analysis, we measured the oxygen consumption rate (OCR) of activated CD8^+^ T cells from both WT and *Ndufs4*^(-/-)^ mice (**Figure 4G**). The basal OCR of *Ndufs4*^(-/-)^ CD8^+^ T cells was comparable to that of WT cells (**Figure 4H**). However, following treatment with FCCP, which uncouples mitochondrial respiration from ATP production, *Ndufs4*^(-/-)^ CD8^+^ T cells showed a marked decrease in maximal OCR in comparison to their WT counterparts (**Figure 4I***; P ≤ 0.01)*, leading to a reduced spare respiratory capacity (SRC) from a mean of 31.13 to 11.12 pmol o2/min (**Figure 4J***; P ≤ 0.01*). These findings indicate that *Ndufs4*^(-/-)^ CD8^+^ T cells are unable to achieve their full respiratory capacity, suggesting impaired ETC in these cells. Overall, our results demonstrate that Ndufs4 deficiency in T cells disrupts CI assembly and OXPHOS, resulting in altered mitochondrial function while mitochondrial biogenesis remains unaffected.

### Increased aerobic glycolysis and reactive oxygen species production in *Ndufs4*^(-/-)^ T cells

**Figure 5:**
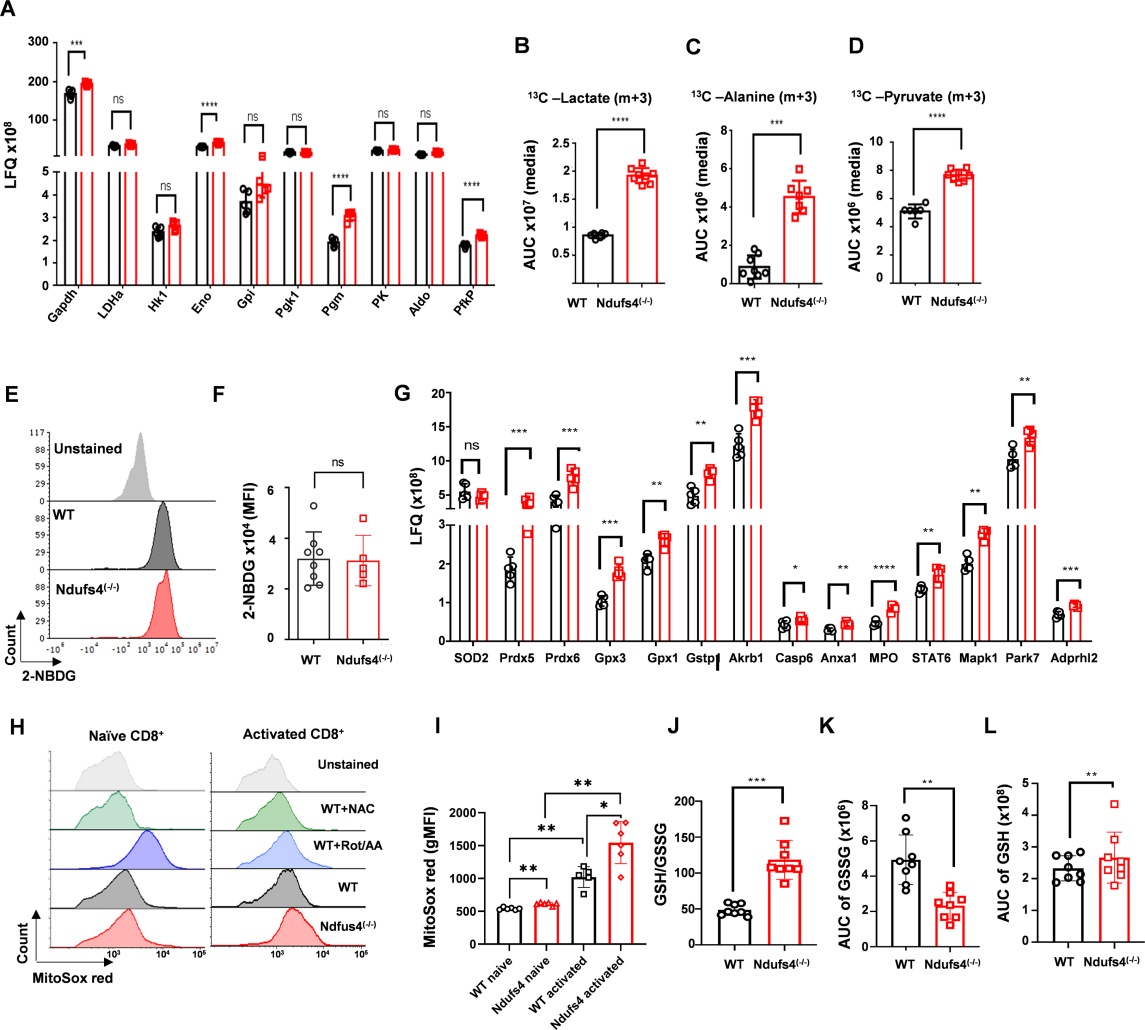
Increased aerobic glycolysis and reactive oxygen species production in *Ndufs4*^(-/-)^ T cells. **(A)** Bar graph showing the Label-Free Quantitation (LFQ) values for glycolysis-related enzymes detected in protein mass spectrometry analysis of WT and *Ndufs4*^(-/-)^ T cells. **(B-D)** Comparison of the area under the curve (AUC) values from metabolomics analysis of ^13^C_6_-glucose tracing, including lactate m+3 **(B)**, alanine m+3 **(C)** and pyruvate m+3 **(D)** from the media fractions. **(E)** Representative flow cytometry histogram of 2NBDG-FITC intensity in WT, and *Ndufs4*^(-/-)^ CD8^+^ T cells shown. **(F)** Bar graph summarizing the mean fluorescence intensity (MFI) of 2NBDG-FITC from **(E, G)** Bar graph displaying LFQ values for ROS detoxification-related enzymes detected in protein mass spectrometry analysis of WT and *Ndufs4*^(-/-)^ T cells. **(H)** Representative flow cytometry histograms of MitoSOX^®^ staining gated on CD8^+^ T cells from naïve or activated splenocytes of WT and Ndufs4^(-/-)^ mice, with WT CD8^+^ T cells used as controls under various conditions: unstained, pre-treated with 200µM N-Acetyl Cysteine (NAC), and treated with rotenone and antimycin A (Rot/AA). **(I)** Bar graph showing the MFI of MitoSOX^®^ from **(H)**. **(J-L)** Bar graphs illustrating the ratio of AUC values for unlabeled glutathione (GSH) versus Glutathione disulfide (GSSG) **(J)**, AUC values for GSSG **(K)**, and GSH **(L)**. Statistical analyses: Student’s t-test was used for protein and glucose-tracing mass spectrometry data. The two-tailed unpaired Mann–Whitney test was used for glucose uptake and MitoSOX^®^ assays. Results are presented as mean ± SD (P values: ns = non-significant; *P ≤ 0.05, **P ≤ 0.01, ***P ≤ 0.001, ****P ≤ 0.0001).

To further understand the possible mechanism underlying the impaired effector functions of *Ndufs4*^(-/-)^ T cells, we followed up on the metabolic rewiring in Ndufs4^(-/-)^ T cells depicted by the protein-set enrichment analysis. For this, we focused on the aerobic glycolysis pathway and the response to oxidative stress. Focusing on glycolysis-related proteins in our proteomic analysis revealed an increase in the expression of 4 of the 10 detected glycolysis-related enzymes in *Ndufs4*^(-/-)^ T cells compared to WT T cells. This includes a significant upregulation of α-enolase (ENO), phosphoglucomutase 2 (PGM), glyceraldehyde 3-phosphate dehydrogenase (GAPDH), and the rate-limiting enzyme phosphofructokinase (PFK) compared to WT T cells (**Figure 5A**). In line with these results, ex-vivo metabolic tracing of ^13^C_6_-glucose using liquid chromatography MS (LCMS)- revealed a significant increase in the levels of lactate m+3, alanine m+3 and pyruvate m+3 in the media (**Figures 5B-D**) of *Ndufs4*^(-/-)^ T cells compared to the control group. Interestingly, evaluating glucose uptake using 2-NBDG-Fitc followed by flow cytometry did not demonstrate a significant increase in glucose uptake by *Ndufs4*^(-/-)^ T cells compared to WT T cells (**Figures 5E, F**; **Supplementary Figures S6A, B**). These findings indicate increased utilization of intracellular glucose for aerobic glycolysis and alanine synthesis by *Ndufs4*^(-/-)^ T cells due to impaired CI activity, leading to OXPHOS inhibition.

Oxidative stress as a result of increased mitochondrial ROS production due to CI dysfunction has been shown previously in fibroblasts from patients with Leigh syndrome induced by pathogenic variants in *Ndufs4 (*15), and in *Ndufs4*-deficient mouse hematopoietic cells (16). Our metabolic analysis showed an increase in various proteins that play a role in redox regulation and ROS detoxification, including glutathione peroxidase 1 and 3 (Gpx1 and Gpx3), glutathione S-transferase 1 (Gstp1), and the thiol-specific antioxidants peroxiredoxin 5 and 6 (Prdx5 and Prdx6) (**Figure 5G**). Next, we sought to directly compare mitochondrial superoxide levels in *Ndufs4*^(-/-)^ T cells and WT T cells using MitoSox staining and flow cytometry. N-Acetyl cysteine (NAC), a glutathione precursor, and antimycin A/rotenone were used as negative and positive controls, respectively, to confirm the validity of the assay. Our results showed that mitochondrial superoxide levels were significantly higher in *Ndufs4*^(-/-)^ CD8^+^ T cells compared with WT cells, already at the naïve state (1.14-fold increase; *P ≤ 0.01*). Following 1 hour of stimulation, this imbalance became even more pronounced, with *Ndufs4*^(-/-)^ CD8^+^ T cells exhibiting a further 1.67-fold increase in ROS production (*P ≤ 0.05*; **Figures 5H, I**). Similar findings were observed in CD4^+^*Ndufs4*^(-/-)^ T cells, with increases of 1.19-fold in the naïve state and 1.63-fold after activation (*P ≤ 0.01* and *P ≤ 0.05*, respectively; **Supplementary Figures S6C, D**).

Increased anti-oxidant activity in Ndufs4^(-/-)^ T cells was further supported by our metabolomic analysis, which demonstrated an increased ratio of reduced to oxidized glutathione (GSH to GSSG ratio; **Figure 5J**), as well as a corresponding increase in GSH and decrease in GSSG levels in Ndufs4^(-/-)^ CD8^+^ T cells (**Figures 5K, L**). Interestingly, metabolomic analysis of naïve CD8^+^ cells demonstrated nearly absent levels of docosahexaenoic acid (DHA) and eicosapentaenoic acid (EPA) in the media of *Ndufs4*^(-/-)^ CD8^+^ T cells (**Supplementary Figures S5E, F**). These omega-3 fatty acids are potent anti-oxidants and their specific absence in the media of *Ndufs4*^(-/-)^ T cells suggests a massive influx into T cells in their attempt to counter ROS activity 

These results clearly show that while *Ndufs4*^(-/-)^ T cells activate various redox regulation and ROS detoxification pathways to compensate for CI dysfunction, they are unable to effectively reduce ROS accumulation, leading to oxidative stress.

### T-cell activation and proliferation are impaired in a patient with Leigh syndrome induced by a loss-of-function point mutation in NDUFS4

**Figure 6:**
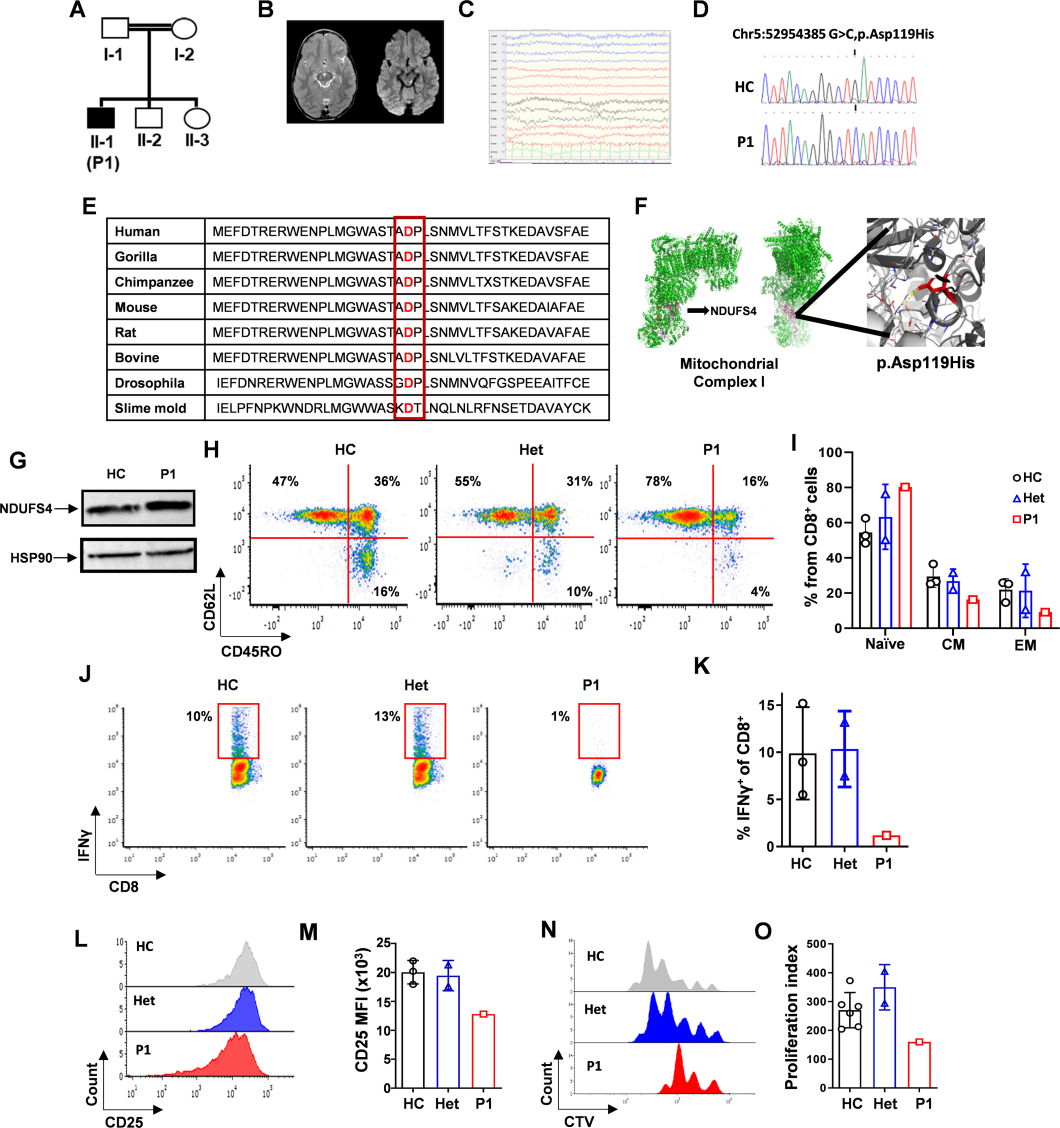
Analysis of a human patient with NDUFS4-defieicnt Leigh syndrome. **(A)** Family pedigree of the patient (P1). Notable is consanguinity with both parents and one sibling heterozygous to the *NDUFS4* variant. **(B)** Brain magnetic resonance imaging (MRI) of P1 with NDUFS4-related Leigh syndrome. Left T2 and right diffusion weighted axial scans through the upper brainstem showing left tectal and right upper brainstem lesions with bright signals of increased T2 and restricted diffusion of acute Leigh syndrome lesions. **(C)** Wake electroencephalogram of P1 at age 5 years showing bilateral moderate slowing of background activity consistent with encephalopathy. **(D)** Chromatogram of DNA sequencing derived from effector T (T_eff_) cells from P1, as compared to a healthy control (HC). A point mutation (NM_002495.4): c.355G>C: p. (Asp119His) in *NDUFS4* can be seen in P1, affecting both alleles. **(E)** P.Asp119His mutation is positioned in a highly evolutionary conserved position of NDUFS4 (16, 17) **(F)** Analysis of a cryo-microscopy model of NDUFS4 (protein data bank: 5XTD (20)) revealing that Asp119 is positioned at the stem of an elongated loop pointing towards the core of CI. **(G)** CD3^+^ T_eff_ cell extracts from HC and P1 were subjected to immunoblot blot analysis using anti-NDUFS4, and anti-HSP90, as loading control. **(H)** Representative flow cytometry plots showing CD45RO vs. CD62L densities gated on CD8^+^ T cells derived from PBMCs of HC, heterozygous parents or P1. **(I)** Bar graph summarizing the results shown in C, as the frequency of naïve (CD45RO^-^ CD62L^+^), central memory (CM, CD45RO^+^ CD62L^+^), and effector memory (EF, CD45RO^+^ CD62L^-^) cells. **(J)** Flow cytometry density plot of CD8 vs. IFNγ, gated on CD8^+^ T cells from PBMCs of P1, his heterozygous parents, and 3 HCs, that were activated with plate-bound CD3/CD28 antibodies for 48 hours **(K)** Bar graph summarizing the results shown in **(L)** Representative flow cytometry stacked histogram of CD25 intensity gated on CD8+ T cells from PBMCs that were activated as mentioned in **(M)** Bar graph summarizing the results shown in L as mean fluorescence intensity (MFI) of CD25 staining. **(N)** Representative stacked histogram of CellTrace violet intensity (CTV) of CD8^+^ T cells that were activated for 5 days with plate-bound CD3/CD28 antibodies. **(O)** Bar graph summarizing the results in N as a proliferation index.

Finally, we aimed to assess the clinical and physiological relevance of NDUFS4 deficiency in human T-cell biology. To do so, we studied a 10-year-old male, referred to as Patient 1 (P1), who was diagnosed with Leigh syndrome at the age of 4. He was born to a consanguineous Sephardic Jewish family with two healthy parents and two healthy siblings. His parents are first-degree cousins (**Figure 6A**).

Leigh syndrome presented at around 1 year of age with delayed motor milestones, ataxia, and tremor. The clinical course was characterized by subacute stepwise deterioration in association with inter-current infections. Brain magnetic resonance imaging (MRI) at 26 months of age supported the diagnosis of encephalopathy and revealed the radiological characteristics of Leigh syndrome (**Figure 6B**). In addition, an electroencephalogram completed at five years of age demonstrated a moderate, generalized, bilateral slowing of background brain activity compatible with diffuse encephalopathy (**Figure 6C**).

A muscle biopsy was obtained at 32 months old and revealed normal striated muscle morphology, whereas biochemical analysis of ETC function revealed decreased function of CI to 37% of control.

P1 has had recurrent fever and was hospitalized with sepsis that was successfully treated with intravenous antibiotics. These infectious events were attributed to his neurological disabilities with resultant aspirations. He did not receive prophylactic antibiotic treatment.

Following this clinical presentation, exome sequencing (ES) was performed to gain a genetic diagnosis. It revealed a homozygous point mutation in *NDUFS4*, (NM_002495.4):c.355G>C:p. (Asp119His) (**Figure 6D**). The variant is in an evolutionarily conserved position (**Figure 6E**) (16, 17).

Since our analysis, others have reported the p.Asp119His variant to be disease-causing (18). Moreover, along with p.Lys154fs, the variant was previously reported to cause Leigh syndrome in a compound heterozygous patient of a non-consanguineous Jewish family. Mitochondrial CI assembly was abnormal in this patient (19). To verify the homozygous p.Asp119His variant in P1, a family segregation study was completed using Sanger sequencing. Both the parents and the male sibling of P1, all of whom are asymptomatic, were found to be heterozygous carriers of the *NDUFS4* variant).

Analysis of a cryomicroscopy model of NDUFS4 (protein data bank: 5XTD) (20) revealed that Asp119 is positioned at the stem of an elongated loop pointing towards the core of CI. In addition, the aspartate side chain has multiple stabilizing hydrogen bonds with core residues in the protein (**Figure 6F**).

Next, we evaluated the expression of NDUFS4 protein. We extracted protein lysates from effector T cells (T_eff_) from P1 and a healthy control (HC). Immunoblot analysis identified a non-diminished, and even increased, level of NDUFS4 protein in T_eff_ cells from P1 compared with an HC (**Figure 6G**). These findings, along with reduced CI activity in the muscle biopsy, suggest that the homozygous p.Asp119His variant leads to LOF of the NDUFS4 subunit of CI without hampering its expression level.

We wanted to reveal possible impairments in P1’s immunity. The complete blood count revealed intact quantities of key myeloid and lymphoid cell subsets (**Supplementary Table S3**; **Supplementary Figure S7A**). The T-cell counts, both CD4^+^ and CD8^+^ T cells, were in the normal range and showed intact TCR diversity compared with the HC (**Supplementary Figures S7A, B**). In-depth immune phenotyping of the T-cell compartment revealed slightly increased ratios of naïve T cells (T_N_), with corresponding reductions in the quantities of memory (T_M_), central memory (T_CM_), and effector memory (T_EM_) cells (**Supplementary Figures S7C**-**E**; **Figures 6H, I**). Regulatory T cells (T_regs_) were within the normal range. The B-cell percentage was slightly decreased; however, humoral immunity appeared to be intact, with normal IgG levels and positive specific IgG antibodies to peptide-containing vaccines, as well as Epstein-Barr virus (**Supplementary Table S3**).

We evaluated T-cell functionality in P1 by measuring cytokine production, the elevation of activation markers, and proliferation upon activation. Flow cytometric analysis of the cells 48 hours after activation with α-CD3/α-CD28 antibodies revealed nearly absent IFN-γ production and decreased CD25 (IL-2 receptor α subunit) surface expression in CD8^+^ T cells from P1 compared with an HC and the heterozygous parents (**Figures 6J-M**). Decreased CD25 expression was also noted in CD4^+^ T cells (**Supplementary Figures S7F, G**). This was followed by analysis of P1’s T-cell proliferation capacity upon activation. The patient’s peripheral blood mononuclear cells (PBMCs) were marked with CellTrace Violet (CTV) and incubated for 5 days in a plate pre-coated with α-CD3/α-CD28. Flow cytometry revealed impaired proliferation of CD8^+^ and CD4^+^ T cells, with a decreased number of T cell generations and an increased count of undivided cells compared with the HC and the heterozygous parents (**Figures 6N, O**; **Supplementary Figures S7H, I**, respectively). In line with the CTV analysis, the number of T_eff_ cells that we retrieved following 14 days of culture was reduced in P1 compared with the HC (41.8 × 10^6^ vs. 100 × 10^6^ cells, respectively).

Impaired T-cell activation and proliferation in P1 could be a result of increased activation-induced cell death (AICD) compared to HCs. To rule out AICD as the cause of P1’s reduced number of effector T cells (T_eff_), we stained T_eff_ cells with propidium iodide (PI). PI staining revealed comparable counts of live CD8^+^ and CD4^+^ T_eff_ cells in P1 and HCs (**Supplementary Figures S7J**-**M**). This further strengthens the conclusion that the decreased number of T_eff_ cells in P1 was the result of impaired cellular proliferation rather than increased cell death.

Taken together, these results suggest that *NDUFS4* LOF has the potential to impair human T-cell–mediated immunity.

## Discussion

In this study, we analyzed the role of Ndufs4, a mitochondrial CI accessory subunit, in T-cell activation and effector functions. We studied a murine T-cell-specific *Ndufs4*^(-/-)^ model and demonstrated impaired T-cell effector functions in *Ndufs4*^(-/-)^ mice via different *in vivo* immunization and adoptive T-cell transfer models.

The first notable finding in *Ndufs4*^(-/-)^ mice was T-cell lymphopenia in the spleen, peripheral blood, and LNs, which was induced by impaired homeostatic expansion, while thymic development was intact and no increased apoptosis was noted in the Ndufs4^(-/-)^ mice. Importantly, thymic development in *Ndufs4*^(-/-)^ mice appeared normal, consistent with a previous study showing that distal LCK deletion, as in our model, does not impair T-cell development in the thymus (21). Using *in vivo* models, we also demonstrated impaired CD4^+^ T-cell-dependent humoral responses and antiviral CD8^+^ T-cell responses in *Ndufs4*^(-/-)^ mice. These findings are consistent with a previous study showing that mitochondrial CI inhibitors, such as rotenone, impair T-cell signaling, activation, and cytokine production (22).

The proteomic and functional analyses revealed that *Ndufs4*^(-/-)^ T cells exhibit a pronounced deficiency in CI subunits, leading to impaired OXPHOS and a marked reduction in spare respiratory capacity. Ndufs4 deficiency has been shown to markedly reduce the abundance of CI subunits (23–25). Accordingly, our results align with previous data regarding the critical role of NDUFS4 in CI assembly and holo-complex formation (26). However, despite this mitochondrial dysfunction, mitochondrial biogenesis and overall content remain intact, as shown by unchanged levels of mitochondrial markers and DNA, as well as preserved mitochondrial morphology.

In response to the energetic deficit caused by CI impairment, *Ndufs4*^(-/-)^ T cells undergo a metabolic rewiring to support increased aerobic glycolysis, as demonstrated by the upregulation of key glycolytic enzymes and elevated production of glycolytic metabolites. This metabolic adaptation mirrors previous reports in other immune cell types, where Ndufs4 deficiency or CI dysfunction triggers a compensatory increase in glycolytic flux to maintain ATP levels ^15^. However, this shift does not fully compensate for the loss of mitochondrial respiratory capacity. Indeed, the absence of Ndufs4 showed to not only impairs CI activity but also to exacerbate the distortion of iron-sulfur clusters, exacerbating CI dysfunction and resulting in elevated mitochondrial reactive oxygen species (ROS) production (26). In line with this, our proteomic and metabolomic analyses show that *Ndufs4*^(-/-)^ T cells upregulate multiple redox-regulatory and antioxidant pathways, including increased glutathione metabolism and expression of detoxifying enzymes. However, these compensatory responses are insufficient to fully neutralize the excess ROS, which contributes to sustained oxidative stress in these cells.

This redox imbalance has broader implications for T cell biology. While ROS are often viewed as damaging byproducts, they also play a pivotal role in T cell signaling and homeostasis. Supporting this, studies in NADPH oxidase (NOX2)-deficient mice have shown that insufficient ROS levels impair Treg function, promote skewed differentiation toward Th1 and Th17 lineages, and drive inflammatory responses (27, 28). This highlights the dual role of ROS in both supporting immune regulation and contributing to pathology when unbalanced.

Our findings in *Ndufs4*^(-/-)^ mice prompted us to explore the physiological relevance of NDUFS4 in human T-cell immunity. Importantly, the T-cell-specific *Ndufs4*^(-/-)^ mice model does not fully replicate the immune phenotype observed in our patient. This is because the patient carries a LOF point mutation, which leads to relatively preserved NDUFS4 protein expression, despite a significant reduction in its function, as seen in the complex I activity in the muscle biopsy. In contrast, the *Ndufs4*^(-/-)^ mice exhibit a complete knockout of NDUFS4 specifically in T cells. Nevertheless, the impaired T-cell activation, evidenced by reduced CD25 upregulation, decreased IFN-γ production, and impaired T-cell proliferation capacity, indicates that NDUFS4 deficiency has physiological relevance in human T-cell biology.

Our recognition that NDUFS4 deficiency impairs T-cell function offers a novel perspective on Leigh syndrome management, especially given that immunological assessment is often overlooked in these patients. Immune profiling may help guide prophylactic antibiotic strategies, revise vaccination plans, and reduce infection risk. Moreover, treatments shown to have efficacy against neurodegeneration in *Ndufs4*^(-/-)^ mice, such as hypoxia and nicotinic acid, may also enhance T-cell immunity in NDUFS4-deficient patients (29). This warrants further clinical investigations in large cohorts of patients with different *NDUFS4* variants.

Our study has several limitations. Notably, it includes only a single patient with *NDUFS4*-related Leigh syndrome. *NDUFS4* variants are an extremely rare cause of Leigh syndrome. According to a systematic review of 195 Leigh syndrome patients with 24 different gene variants, only 22 cases involving *NDUFS4* mutations have been reported worldwide (30). In addition, a previous study have demonstrated residual CI activity in *Ndufs4*^(-/-)^ mice through CI-CIII super-complex formation (31). However, we did not examine whether CI-CIII super-complexes form in T cells lacking Ndufs4 to compensate for its absence, which was beyond the scope of our study.

In summary, Ndufs4 deficiency impairs mitochondrial energetics and causes redox imbalance in T cells, leading to maladaptive metabolic reprogramming and sustained oxidative stress. Our findings underscore the essential role of complex I activity and ROS regulation in T-cell metabolism and immunity, and suggest that targeting metabolic and redox pathways may be a promising strategy in diseases involving mitochondrial dysfunction in immune cells.

## Materials and methods

### Murine model

#### Breeding of T-cell specific NDUFS4 ^(-/-)^ mice

B6.129S4-Ndufs4tm1Rpa/J 5-week old mice were purchased from the Jackson Laboratory (Stock No: 026963) (32) (32). All experiments, excluding thymus phenotyping, were conducted using 10-15-week old mice. Our study examined male and female animals, and similar findings are reported for both sexes.

*Ndufs4*^(loxp/loxp)^ mice were cross-bred with distal (d) Lck-Cre mice, which resulted in excision of exon 2 of the Ndufs4 gene specifically in T cells. The dLck-Cre 3779 founder line purchased from the Jackson Laboratory is highly T cell–specific, with insignificant recombination in B cells, NK cells, or myeloid cells (33). *Ndufs4*-deficient mice were backcrossed to a pure C57BL/6 background. Differentiation between wild type (WT) and *Ndufs4*^(loxp/loxp)^ mice was done by polymerase chain reaction (PCR; **Supplementary Figures S2D, E** for Lck-cre^+^ and *Ndufs4*^(loxp/loxp)^ mice, respectively). Moreover, to validate Ndufs4 knockout, we have designed specific primers to detect *Ndufs4*^(-/-)^ following excision of exon 2 by the Lck-cre recombinase.

In addition, we have cross-bred OT-I mice (C57BL/6-Tg (TcraTcrb) 1100Mjb/J (OT-I) mice (strain#003831), and B6.SJL-Ptprca Pepcb/BoyJ mice (CD45.1); The Jackson laboratories) with *Ndufs4*^(-/-)^ mice to create OT-I/*Ndufs4*^(-/-)^ mice. OT-I mice are transgenic for TCR Vα2 and Vβ, thus allowing their CD8^+^ T cells to recognize the SIINFEKL peptide of the ovalbumin (OVA) protein (34). All animal studies were approved by the Institutional Animal Care and Use Committee of the Hebrew University, Jerusalem, Israel.

PCR Primers used for mice genotyping are detailed in **Supplementary Table S1**.

#### Mice anesthesia and euthanasia

Female and male mice (20–30 g) were euthanized in accordance with approved institutional animal ethics protocols. Mice were first anesthetized by intraperitoneal injection of ketamine (200 mg/kg) and xylazine (10 mg/kg). Once adequate anesthesia was confirmed, euthanasia was performed by cervical dislocation.

#### Immunoblotting of NDUFS4

To confirm knockout of NDUFS4, CD3^+^ T cells were separated from splenocytes using an immunomagnetic negative selection-based cell isolation kit (StemCell™ technologies, Vancouver, Canada; #19851A). CD3^+^ T cells were then lysed and transferred into a nitrocellulose membrane, as explained before. Skim milk in a concentration of 5% and diluted in TBST was used for blocking. Following blocking, we used anti-Ndufs4 probe (NOVUS, USA; # NBP1-31465) to detect the Ndufs4 protein. Anti-β-tubulin antibody (Santa Cruz Biotechnology, Texas, USA; #sc-5274) used a loading control.

#### Flow-cytometry analysis of lymphocyte subsets

Flow cytometry was used for immune phenotyping of lymphocytes. NDUFS4 ^(-/-)^ mice were sacrificed and splenocytes, thymocytes, lymph node lymphocytes and PBMC were harvested, filtered through cell strainers, washed with FCS and stained with the following antibodies:

Splenocytes- CD8 (PB; #53-6.7), CD4 (Alexa Fluor 700;#GK1.5), CD44 (FITC; #IM7), CD122 (PE; #5H4), CD127 (APC; #A7R34) and CD62L (PE/CY7; #MEL-14); Thymocytes- TCRβ (FITC; #H57-597), CD4 (PE; #GK1.5), CD69 (PE/CY7; #H1.2F3), CD8 (PB; #53-6.7) and CD24 (APC; #30-F1); PBMC- CD45.2 (PB; #104), CD19 (PE; #6D5) and CD8 (FITC; #53-6.7); Lymph nodes- CD90.2 (PE/Cy7; #30-H12), CD4 (Alexa Fluor 700; #GK1.5) and CD8 (PB; #53-6.7).

For *in-vivo* experiments, we used CD44 (APC/Cy7; #IM7) and CD62L (Pe/Cy7; #MEL-14). CD45.1 (FITC; #A20) and CD45.2 (APC; #104) were used to differentiate between donor and recipient mice in adoptive T-cell transfer experiments. Readout of flow-cytometry was analyzed using FCS Express™, version 6, *De-Novo* software, California, USA. Gating strategies for flow-cytometry analyses of **Figures 1**-**3**, **5**, **6** are presented in **Supplementary Figures S8**-**11**.

#### *In-vivo* homeostatic expansion assay

To examine homeostatic expansion, adoptive T-cell transfer was employed. Donor mice were differentiated between WT and Ndufs4^(-/-)^ by the CD45 marker (CD45.1^+^ and CD45.2^+^, respectively). Recipient mice were on a pure C57BL/6 background. Initially, recipient heterozygous CD45.1^+^/CD45.2^+^ WT mice were sub-lethally irradiated (600 rad). The following day, each recipient mouse was intravenously injected with 200 µL of PBS containing a CellTrace (Invitrogen, Massachusetts, USA; #2683566)-labeled mixture of splenocytes, normalized to deliver a total of 8 x 10^6^ T cells at a 1:1 ratio of WT to Ndufs4^(-/-)^ genotypes. After 7 days, quantification of naïve T cells (T_N_) from WT and *Ndufs4*^(-/-)^ donor mice and the signal of CellTrace, indicative of T_N_ homeostatic expansion, were measured using flow cytometry. Proliferation index for each CellTrace-based proliferation assay was calculated by multiplying the percentage of divided cells in each division by the corresponding generation number, with undivided cells (the first peak) assigned a value of 0. The proliferation index represents the sum of all these products, providing an integrated measure of total proliferative activity.

#### *In-vivo* immunization model to analyze CD4^+^ T-cell activation

WT and Ndufs4^(-/-)^ mice were subcutaneously injected with 100 µg/100µL PBS of OVA protein (Sigma Aldrich, Massachusetts, USA; #9006-59-1) along with complete Freund adjuvant (CFA; Sigma Aldrich, Massachusetts, USA; #F5881) over a span of two weeks, with one injection per week. This was followed by a booster injection containing OVA alone in the third week. After a 3-week inoculation period, the mice were euthanized, spleens were extracted for immunofluorescence microscopy, and serum samples were collected for enzyme-linked immunosorbent assay (ELISA).

#### ELISA analysis of cytokines and antibodies in serum

ELISA was used to quantify cytokines and antibodies in the sera of mice. Investigated cytokines consisted of interferon (IFN)-γ (# BLG- 430804) and IL-4 (#BLG-431104); Biolegend, California, USA. ELISA assays to detect cytokines were conducted according to the manufacturer’s protocols.

In addition, antibodies were quantified using ELISA kits for IgG1 (#1144-05) and IgG2a (#1155-05); Biolegend, California, USA. For IgG1 and IgG2a, plates were pre-coated with OVA in a coating buffer containing 50mM Na2CO3, 50mM NaHCO3, NAN3 0.1%, PH = 9.6 and 1L DDW. Plates were then blocked with Phosphate Buffered saline (PBS) containing BSA 3%. This was followed by 3 washings with 1X Phosphate-Buffered Saline, 0.1% Tween (PBST). Diluted sera from the studied mice (dilutions of 10^-1^-10^-5^) were added to the plate. Next, secondary antibody diluted 1:5000 in PBST was added to each well. Finally, after 3 washings, Tetramethylbenzidine (TMB) was added, and readout of the plate was conducted using a plate reader (wavelength 650 nm).

#### Immunofluorescence

Stainings were performed as described earlier (35). Briefly, spleen tissues were embedded in Optimal Cutting Temperature (OCT) compound (Scigen O.C.T. Compound Cryostat Embedding Medium, 23-730-625) and sectioned in a cryostat to 7µm sections. Sections were then fixed with 4% PFA, blocked using 3% BSA, 3% goat serum and 0.03% Tween-20 and stained with antibodies against CD3ϵ (PE) CD11b (APC), and CD79b (FITC) (all from Biolegend, California, USA). Subsequently, sections were stained with DAPI (#D1306) and mounted using ProLong Gold mounting medium (#P10144); both purchased from Thermo Fisher Scientific, Massachusetts, USA. Images were taken using an Olympus IX83 Inverted Microscope.

#### Adoptive T-cell transfer for *in-vivo* study of T-cell proliferation

To analyze T-cell proliferation capacity *in-vivo*, splenocytes from OT-I/WT and OT-I/Ndufs4 ^(-/-)^ donor mice were harvested and then treated with CellTrace violet (Invitrogen, Massachusetts, USA; #2683566). Thereafter, 4 x 10^6^ CellTrace-labeled splenocytes/200µL PBS were intravenously injected to recipient mice. The next day, recipient mice were treated with OVA by intraperitoneal injections. Finally, on day 3 to the experiment, recipient mice were sacrificed, splenocytes were harvested and subjected to surface staining with antibodies to CD4 (PE,GK1.5), CD8 (FITC, 3-6.7) and CD25 (APC; PC61), followed by flow cytometry analysis of the CellTrace signaling.

#### *In-vivo* model of Lentivirus-OVA-induced T-cell activation

To test CD8^+^ T-cell activation, we used a model of infection with Lentivirus-OVA. Lentivirus-OVA-GFP was a kind gift granted by Prof. Avi-Hai Hovav, form the Hebrew University, Jerusalem, Israel. Lentivirus-OVA-GFP in a dose of 5 x 10^6^ transfection units (TU) was intradermally injected to the left ear pinna of OT-I/WT and OT-I/*Ndufs4*^(-/-)^ mice. Following one week, left cervical lymph nodes were extracted and T cells were surface stained for effector markers, including CD8, Vα2, CD44, CD25 and CD62L, thus quantification of CD8^+^ T_eff_ was achieved using flow cytometry. Right ear pinna did not receive the Lentivirus-OVA-GFP. Therefore, right cervical lymph nodes were used as a negative control.

### Assessment of mitochondrial functions

#### Measurement of oxygen consumption rate

CD8^+^ T cells were separated from splenocytes using an immunomagnetic negative selection-based cell isolation kit (StemCell ™ technologies, Vancouver, Canada, #100013988). Oxygen consumption rate (OCR) was analyzed in purified naive CD8^+^ T cells of WT and Ndufs4 ^(-/-)^ mice, using a standard Seahorse Xfe96 analysis. Detailed methodology for Seahorse analysis was previously described by Saragovi et al. (7). In brief, 4 x 10^5^ naïve CD8^+^ T cells were tested per well and OCR, including basal OCR, OCR during the assay period and maximal OCR were measured. During the Seahorse study, CD8^+^ T cells were treated at different time points with oligomycin, rotenone and antimycin-A (1µM, each), which inhibit mitochondrial CV (ATP synthase), CI and CIII, respectively. CD8^+^ T cells were also treated with 2µM carbonyl cyanide-p-trifluoromethoxyphenylhydrazone (FCCP), a known mitochondrial OXPHOS uncoupler. Spared respiratory capacity (SRC) was defined by the difference between the maximal OCR following FCCP treatment and basal OCR.

#### Quantification of mitochondrial DNA content of CD8^+^ T cells using real-time PCR

CD8^+^ T cells were separated from splenocytes of WT and NDUFS ^(-/-)^ mice using an immunomagnetic negative selection-based cell isolation kit, as explained earlier.

Total DNA, encompassing nDNA and MtDNA, was purified from CD8^+^ T cells through the following steps: T cells were lysed overnight at 55°C using a lysis buffer comprising 10 mM Tris-HCl (pH=8), 1 mM EDTA, and 0.5% SDS. Proteinase K was added at a ratio of 1:10 to the lysis buffer. On the second day, samples were vigorously vortexed, followed by centrifugation for 15 minutes at 8,000G at room temperature. After centrifugation, supernatants were transferred to a new tube, and the pellet was discarded. Phenol/chloroform/isoamyl alcohol (25:24:1) was added and mixed using a spinning device for 5 minutes at 50–60 RPM to ensure proper layer mixing. After centrifugation for 15 minutes at 8000G, the layers were separated, and the upper DNA layer was collected. The supernatants were transferred to a new tube, followed by the addition of an equal volume of chloroform. Subsequent centrifugation for 15 minutes at 8,000G was performed, followed by the addition of 3M NaAc and cold isopropanol. Samples were then maintained at -20°C for 10 minutes for DNA precipitation. Following centrifugation for 15 minutes at maximum speed, the supernatants were discarded, and the DNA pellets were washed with 1 ml of 70% cold ethanol. After another centrifugation step, the supernatants were discarded, and the pellets were air-dried. Subsequently, 40 µl of DDW was added. A DNA concentration of 200 ng/µL was used to quantify mtDNA. Finally, the ratio between the DNA of Mito-RNR2 and β2 microglobulin (β2M), representative genes of MtDNA and nDNA, respectively, was completed using semi-quantitative (sq) real-time (RT)-PCR. Primers used for sqRT-PCR are presented in **Supplementary Table S2**.

#### Confocal microscopy of T-cell mitochondria

WT and *Ndufs4*^(-/-)^ mice were sacrificed, and their spleens were collected. CD8^+^ T cells were then isolated from the splenocytes using the previously described method. Naïve CD8^+^ T cells were stained with deep red MitoTracker^®^ mitochondrion-selective probes (Invitrogen, Massachusetts, USA; #M22426, ex: 644, Em: 665), following the manufacturer’s protocol (37). A 20nM working solution was prepared by diluting the 1mM stock solution in pre-warmed Hanks’ Balanced Salt Solution (HBSS). One million CD8^+^ T cells were resuspended in 200 µl of this working solution and incubated at 37°C for 45 minutes in the dark. After incubation, the cells were washed with pre-warmed PBS or HBSS, and the supernatant was discarded. The stained CD8^+^ T cells were then placed into a high-resolution chamber (1.5H, with a thickness of 170µm ± 5µm) from Ibidi (# IBD-80807) in FACS buffer. Imaging was performed using a Zeiss LSM 980 with Airyscan technology on a Zeiss Axio Observer 7 SP inverted microscope with a Plan-Apochromate 63×/1.4 Oil DIC objective. Data analysis was carried out with NIS-Elements software from Nikon Instruments, Amstelveen, Netherlands.

#### Protein mass spectrometry using liquid chromatography

The liquid chromatography-mass spectrometry (LC-MS) analysis was performed at the Stein Family Mass Spectrometry Center, located in the Alexander Silberman Institute of Life Sciences at the Hebrew University of Jerusalem, Givat-Ram, Israel.

#### Sample preparation

OT-I/WT and OT-I/*Ndufs4*^(-/-)^ mice were sacrificed, and spleens were harvested. Naive CD8^+^ T cells were then separated from the splenocytes using an immunomagnetic negative selection-based cell isolation kit (StemCell™ technologies, Vancouver, Canada, #100013988). Each sample included 1.5x 10^6^ CD8^+^ T cells. Cells were washed twice with PBS, centrifuged with 17000 G for 3 minutes, followed by removal of supernatants. Cells were then lysed in 25 mM Tris-HCl pH 8.0 containing 4% sodium dodecyl sulfate. The soluble proteins were reduced by the addition of 10 mM dithiothreitol for 30 min and alkylated by addition of 55 mM iodoacetamide (Sigma Chem. Corp. St. Louis, MO) and incubation for 30 min. at room temperature in the dark. Removal of SDS followed by digestion with sequencing grade modified trypsin (Promega Corp., Madison, WS) were performed using the S-Trap microspin column kit as specified by the manufacturer (Protifi, LLC, Huntington, NY). The tryptic peptides were desalted on home-made C18 Stage tips. A total of 0.16 µg of peptides (determined by Absorbance at 280 nm) from each sample were injected into the mass spectrometer.

#### Nano liquid chromatography–mass spectrometry/mass spectrometry analysis

MS analysis was performed using a Q Exactive-HF mass spectrometer (Thermo Fisher Scientific, Waltham, MA USA) coupled on-line to a nanoflow ultra-high-performance liquid chromatography (UHPLC) instrument, Ultimate 3000 Dionex (Thermo Fisher Scientific, Waltham, MA USA). Peptides dissolved in 0.1% formic acid were separated without a trap column over a 100 min acetonitrile gradient (1 – 80% acetonitrile), run at a flow rate of 0.3 μl/min on a reverse phase 25-cm-long C18 column (75 μm ID, 2 μm, 100Å, Thermo PepMapRSLC). The instrument settings were as described by Scheltema et al. (36). Survey scans (300–1,650 m/z, target value 3E6 charges, maximum ion injection time 20 ms) were acquired and followed by higher energy collisional dissociation (HCD) based fragmentation (normalized collision energy 27). A resolution of 60,000 was used for survey scans and up to 15 dynamically chosen most abundant precursor ions, with “peptide preferable” profile was fragmented (isolation window 1.6 m/z). The MS/MS scans were acquired at a resolution of 15,000 (target value 1E5 charges, maximum ion injection times 25 ms). Dynamic exclusion was 20 sec. Data were acquired using Xcalibur software (Thermo Scientific). To avoid a carryover, the column was washed with 80% acetonitrile, 0.1% formic acid for 25 min between samples.

#### Mass spectrometry data analysis

Mass spectra data were processed using the MaxQuant computational platform, version 1.6.17.0. Peak lists were searched against mouse proteome database from Uniprot, containing 36,759 sequences. The search included cysteine carbamidomethylation as a fixed modification, N-terminal acetylation and oxidation of methionine as variable modifications and allowed up to two miscleavages. The ‘match-between-runs’ and the ‘dependent peptide’ options were used. Peptides with a length of at least seven amino-acids were considered and the required FDR was set to 1% at the peptide and protein level. Relative protein quantification in MaxQuant was performed using the label-free quantification (LFQ) algorithm (37).

#### Glucose-tracing metabolome

A glucose-tracing metabolome study was conducted at the Ruth and Bruce Rappaport Faculty of Medicine, Technion- Israel Institute of Technology, Haifa, Israel.

#### Sample preparation

Naïve CD8^+^ T cells from OT-I/WT and OT-I/*Ndufs4*^(-/-)^ mice were initially purified through negative selection following standard procedures. These cells were then exposed to 11mM ^13^C_6_-Glucose for 4 hours. To extract metabolites, we mixed 40 µl of culture medium with 40 µl of a chilled extraction solution (−20°C), containing methanol, acetonitrile, and water in a 5:3:2 ratio. The cells underwent three rapid washes with ice-cold PBS, and metabolites were extracted using 100 µl of the cold extraction solution for 5 minutes at 4°C, followed by three freeze-thaw cycles. The media and cell extracts were centrifuged at 17,000 g for 10 minutes at 4°C to eliminate insoluble particles, and the supernatant was then analyzed by LC-MS. The metabolomics data were normalized to protein concentrations using the Bradford assay.

#### Liquid chromatography–mass spectrometry analysis

For the analysis, both the media and cell extract supernatants were examined using a Thermo Ultimate 3000 HPLC system linked to a Q-Exactive Orbitrap Mass Spectrometer (Thermo Fisher Scientific). The mass spectrometer operated with a resolution of 35,000 at a 200 mass/charge ratio (m/z) and utilized electrospray ionization with polarity switching to capture both positive and negative ions across a mass range of 67 to 1000 m/z.

#### High-performance liquid chromatography setup

The high-performance liquid chromatography (HPLC) system was configured with a ZIC-pHILIC column (SeQuant; 150 mm x 2.1 mm, 5 µm; Merck) and a ZIC-pHILIC guard column (SeQuant; 20 mm x 2.1 mm). A 5 µl aliquot of biological extracts was injected into the system. Compound separation was achieved using a 15-minute gradient mobile phase, beginning with 20% aqueous (20 mM ammonium carbonate adjusted to pH 2 with 0.1% of 25% ammonium hydroxide) and 80% organic (acetonitrile), and ending with 20% acetonitrile. The flow rate was set to 0.2 ml/min and the column temperature was maintained at 45°C, resulting in a total analysis time of 27 minutes.

#### Analysis of data gathered by mass spectrometry

All metabolites were identified with a mass accuracy of less than five parts per million (ppm). Thermo Xcalibur facilitated data acquisition, while analysis occurred via TraceFinder 4.1. Peak areas of metabolites were determined utilizing the exact mass of singly charged ions, and the retention time of metabolites was pre-established on the pHILIC column through analysis of an in-house mass spectrometry metabolite library constructed by running commercially available standards.

#### Glucose uptake assay

To evaluate glucose uptake, T_N_ cells were incubated for 30 minutes with medium containing 60µM of the fluorescent probe 2-deoxy-2-D-glucose (2-NBDG; FITC). Following incubation, 2-NBDG signal was read by flow cytometry.

#### MitoSOX ™ staining of ROS in naïve and activated T cells

WT and *Ndufs*4^(-/-)^ mice were sacrificed, and their spleens harvested. This was followed by separation of CD8^+^ T cells using a negative isolation kit, as detailed above. Naïve and 1-hour activated CD8^+^ T cells were pre-heated with HBSS at 37°C. Activation of CD8^+^ T cells was done with suspended ultra-LEAF™ anti-CD3ϵ (#145-2C11) and anti-CD28 (#37.51) antibodies in a dose of 400ng/mL (both purchased from Biolegend, California, USA). This was followed by incubation of 1×10^6^ cells per well (96 flat-bottom well plate) with 1µM of MitoSOX ™ (Invitrogen, Massachusetts, USA; #M36008) for 30 minutes. Finally, cells were washed three times with preheated PBS and centrifuged each time at 300 g for 5 min at room temperature. Readout of the dye signal was done by flow cytometry.

#### Statistical analysis

Two-tailed Mann-Whitney non-parametric, unpaired test and Student’s T-test were performed using GraphPad Prism version 6.0.0 for Windows, GraphPad Software, Boston, Massachusetts USA. P-value ≤ 0.05 was considered statistically significant. Metascape comparative analysis was used to examine alterations in the cellular proteome (38). In addition, Perseus software platform version 1.6.13.0. was used for proteome and metabolome quantifications (39).

### Human study

#### A patient with Leigh syndrome induced by a NDUFS4 loss-of-function variant

A patient with NDUFS4-related LS (P1) was diagnosed and treated at the pediatric neurology unit of Hadassah Medical Center, Jerusalem, Israel. The patient was treated during the period of 2015-2021. The study was approved by the institutional review board (IRB) of Hadassah Medical Center (IRB number: 0024-19-HMO). Peripheral blood was drawn following signing informed consent by the patient’s parents and according to the IRB guidelines.

#### Genetic confirmation of mitochondrial variant

P1 was suspected to have underlying genetic disease and was diagnosed with a singleton exome sequencing (ES) including mtDNA sequencing conducted at Hadassah Medical Center, as previously described (40). Based on the parental consanguinity and the decrease of mitochondrial CI activity in muscle tissue, exonic variants were filtered based on zygosity (homozygous) and localization in genes encoding CI assembly components.

#### Lymphocyte subpopulation analysis

Peripheral blood mononuclear cells (PBMC) were separated from whole blood using Lymphoprep^®^ separation kit, according to the manufacturer instructions (16, 17). Cells were then subjected to surface staining using antibodies (all purchased from Biolegend, California, USA) to CD3 (APC; #SK7), CD4 (APC; #OKT4), CD8 (PB; #SK4), CD19 (FITC; #HIB19), CD56 (PE; #HCD56), CD45RA (FITC; #H1100), CD45RO (PE; #UCHL1) and CD62L (Alexa Fluor 700; #DREG-56).

Methodological details regarding T_regs_ quantification was previously described (41). In brief, PBMC of P1 and healthy controls (HC) were surface stained for CD4 (APC; #OKT4), and CD25 (Alexa Fluor 700; #BC96) and then fixated and perambulated using FoxP3 staining buffer set, according to the manufacturer’s protocol (Invitrogen, Massachusetts, USA). This was followed by intra-nuclear staining for forkhead box protein 3 (FOXP3)^+^, using anti-FOXP3 antibody (PE; 206D; Miltenyi Biotec, Auburn, California, USA).

Age-matched references values were taken from Jolliff et al. (42) and Garcia-Prat et al. (43). Data retrieved from flow cytometry were analyzed using FCS Express™, version 6, *De-Novo* software, California, USA.

#### Evaluation of T cell activation

For assessment of T-cell activation, PBMC were incubated for 48 hours in a 96-well flat bottom plate. The plate was pre-coated with boric buffer containing α-CD3/CD28 ultra-LEAF antibodies (#OKT3 and #CD28.2, respectively; both purchased from Biolegend, California, USA) in a concentration of 6μg/mL. PBMC were incubated within a standard RPMI 1640 medium with addition of 10% fetal calf serum (FCS), 2 mM L-glutamine, 100 U/mL penicillin and 0.1 mg/mL streptomycin (PSS) and 400 U/mL of recombinant human interleukin (IL)-2. Following 48 hours of incubation lymphocytes were subjected to surface staining with CD8 (PB; #SK1) and CD25 (Alexa Fluor 700; #BC96) antibodies. T_eff_ cell functions were assessed by flow-cytometry following intracellular staining with leucoperm^®^ standard kit (Bio-Rad Laboratories, California, USA; # BUF09C) and anti-interferon γ (IFNγ) antibody (BD, New Jersey, USA; #4S.B3). As exogenous IL-2 was added to the media, we did not measure its production by T cells. T cells of healthy controls (HC), which were examined for activation and proliferation assays, were non-age-matched. Therefore, Naïve and memory T cells from both P1 and the HC were quantified before activation to verify comparable counts of cells.

#### Measurements of T-cell proliferation capacity

To evaluate T-cell proliferation capacity, PBMC were separated from whole blood and treated with CellTrace violet (Invitrogen, Massachusetts, USA; #2683566). Indicated PBMC were then incubated for 5 days in a 96-well flat bottom plate, pre-coated with boric buffer containing α-CD3/CD28 antibodies, as mentioned above. Following 5 days of incubation, T cells were harvested and subjected to surface staining with antibodies to CD4 (APC; #OKT4), CD8 (PE; #HITC) and CD25 (Alexa Fluor 700; BC96), followed by flow cytometry analysis of the CellTrace signaling.

#### Effector T cells cultures

Due to limited availability of the patient’s blood samples, we utilized T_eff_-cell cultures for more advanced studies. PBMC were incubated in a 25 mL flask with RPMI 1640 containing 10% FCS, 2 mM glutamine, 55 µM 2-mercapthoethanol, 25 mM HEPES buffer solution and PSS. A-CD3 antibody and recombinant IL-2 were added to the media to a final concentration of 0.02 µg/mL and 6000 U/mL, respectively. T_eff_ cells were relocated to a 75 mL flask and harvested and counted, following 8 and 14 days of incubation, respectively. Harvested T_eff_ cells were than stained for CD4 (APC; #OKT4) and CD8 (PB; #SK1). Furthermore, to verify T_eff_ survival percentages and exclude activation-induced cell death (AICD) we have used flow cytometry to quantify dead T cells marked with propidium iodide solution (PI; PE; Sigma-Aldrich, Missouri, USA; #MKCM8441).

#### TCR V-β repertoire

The analysis of TCR V-β expression was conducted at the primary immune deficiency laboratory, Tel-Hashomer Medical Center, Ramat-Gan, Israel. It was determined for P1 according to the manufacturer’s manual (Beta Mark TCR Vβ Repertoire Kit, Beckman Coulter), as previously detailed (41).

#### Immunoblotting of NDUFS4

T_eff_ cells were lysed using radioimmunoprecipitation assay (RIPA) buffer containing protease inhibitor (APExBIO; catalog number: K1007). Protein lysates were then subjected to sonication (2 minutes, 70% amplitude) and blotted into a nitrocellulose membrane. Skim milk in a concentration of 5% and diluted in Tris-buffered saline with Tween Detergent (TBST) was used for blocking. Following blocking, we used an anti-NDUFS4 probe (Abcam, Cambridge, United Kingdom; #ab137064) to detect the NDUFS4 protein. Anti-heat-shock protein (HSP) 90 antibody (Cell signaling, Massachusetts, USA; #4874S) was used as a loading control.

## Data Availability

The raw data supporting the conclusions of this article will be made available by the authors, without undue reservation.

## Supplementary material

Supplementary Figure 1 T-cell-specific Ndufs4(-/-) mice model. **(A)** Ndufs4(loxp/loxp) mice were cross–bred with distal (d)Lck-Cre+ mice. The Cre recombinase excised exon 2 in the Ndufs4 gene, specifically in T cells. **(B)** To confirm knockout of Ndufs4, polymerase chain reaction (PCR) using primers designed to amplify the Ndufs4 DNA following the excision of exon 2 by the Cre recombinase was utilized. Thus, enabling differentiation of Ndufs4(-/-) from other mice. **(C)** Naïve CD3+ T cells were separated from splenocytes of Ndufs4(-/-) and WT mice using an immunomagnetic negative selection-based cell isolation kit. CD3+ T cells were then lysed and transferred to a nitrocellulose membrane. Following blocking, an anti-Ndufs4 probe was used to detect the Ndufs4 protein. Anti-β-tubulin antibody was used in the immunoblotting as a loading control. **(D)** Representative PCR of Cre+ mice. Myogenin is used as a DNA loading control. **(E)** Representative PCR of Ndufs4(loxp/loxp) mice.

Supplementary Figure 2 Immune phenotyping of peripheral blood, lymph nodes, and spleens of Ndufs4(-/-) mice. **(A)** Peripheral blood mononuclear cells (PBMC) of Ndufs4(-/-) and WT mice were stained for CD19 and CD3. Bar graph summarizing the results (n=6 mice, for each group). **(B)** Ndufs4(-/-) and WT mice were sacrificed, and cervical lymph nodes were extracted and stained for lymphocyte markers. Bar graph summarizing results of flow-cytometry analysis of CD90, a pan-T cell marker (n=4 and 6 mice for WT and Ndufs4(-/-)mice, respectively). **(C)** Representative flow-cytometry of CD4+ and CD8+ T cells derived from cervical lymph nodes of Ndufs4(-/-) and WT mice. **(D)** Bar graph summarizing results of C (n=6 mice, for each group). **(E)** Representative flow cytometry plots showing the frequencies of naïve (CD44loCD62Lhi), TCM (CD44hiCD62Lhi), and TEM (CD44hiCD62Llo) subsets of CD8+ T cells in the spleens derived from WT and Ndufs4(-/-) littermate mice. **(F)** Bar graph summarizing the results of E, indicating percentages (n=6 mice, for each group). **(I)** Same as H, summarizing absolute cell numbers of CD8+CD44hiCD122hi T cells. (n=6 mice, for each group). **(G, H)** Same as **(E, F)**, respectively, for the CD44loCD122hi subset of CD8+ T cells. (n=6 mice, for each group). Statistical method: two-tailed unpaired Mann–Whitney test. Results are presented as the mean values ± SD (P value, ns-non-significant; *P≤0.05, **P≤0.01, *** P≤0.001).

Supplementary Figure 3 Intact thymic subsets and impaired homeostatic expansion of CD4+ T cells is seen in Ndufs4(-/-) mice. **(A)** Representative flow cytometry plots demonstrating the frequencies of single positive (CD4+ or CD8+), double positive (CD4+CD8+), and double negative (CD4-CD8-) thymocytes derived from 5-week-old Ndufs4(-/-) and WT littermate mice. **(B)** Bar graph summarizing the results of A (n=5 mice, for each group). **(C)** Representative flow cytometry plots of CD45.1 vs. CD45.2 staining of CD4+ splenocytes from recipient mice, 7 days after cell transfer. The Homeostatic expansion experiment was previously detailed in **Figure 6**. **(D)** Bar graph summarizing the results shown in C. **(E)** Representative flow cytometry stacked histogram of Cell-Trace violet (CTV) intensity of donor CD4+ T cells. **(F)** Bar graph summarizing the results shown in E, as proliferation index (n=6 mice, for each group). **(G)** Representative flow cytometry plots demonstrating the frequencies of Propidium iodide (PI)+ Annexin V- **(A)**, PI-Annexin V- **(B)**, PI- Annexin V+ **(C)**, and PI+ Annexin V+ **(D)** CD8+ splenocytes from WT and Ndufs4 (-/-) mice. **(H)** Bar graph summarizing the results of A. Statistical method: two-tailed unpaired Mann Whitney test. Results are presented as the mean values ± SD (P value, ns- non-significant, **P≤0.01).

Supplementary Figure 4 Controls for the in-vivo infection model used to study CD8+ T cell activation. **(A)** In-vivo model examining CD8+ T-cell effector functions. Lv-OVA was injected to the left ear pinnae of Ndufs4(-/-) and WT littermate mice. Seven days later, left cervical lymph nodes were extracted and analyzed by flow cytometry; right cervical lymph nodes were used as negative controls, as right ear pinnae of each mouse were not exposed to the Lv-OVA. **(B)** Bar graph comparing cell counts of right and left cervical lymph nodes in Ndufs4(-/-) and WT littermate mice (n=5 mice, for each group). **(C, D)** Same as **(B)**, with counts of CD8+ and CD8+Vα2+ T cells, respectively (n=5 mice, for each group). **(E)** Bar graph summarizing cell counts of CD4+ and CD8+ T cells derived from right cervical lymph nodes of each Ndufs4 (-/-) and WT littermate mice (n=5 mice, for each group). Statistical method: two-tailed unpaired Mann–Whitney test. Results are presented as the mean values ± SD (P value, ns-non-significant; *P≤0.05, **P≤0.01).

Supplementary Figure 5 Confocal microscopy of mitochondria in naïve Ndufs4 (-/-) T cells. **(A)** Ndufs4 (-/-) and WT littermate mice were sacrificed, and their spleens were harvested. CD8+ T cells were separated using an immunomagnetic negative selection kit, and a mixture was prepared for each mouse group. Mitochondria were then stained with MitoTracker® far-red and visualized by confocal microscopy (n=3 mice, for each group). **(B-E)** Dot plots summarizing mitochondrial intensity, circularity, length, and surface area, respectively, of CD8+ T cells derived from Ndufs4 and WT littermate mice. Each dot in the graphs represents a single mitochondrion. Statistical method: two-tailed unpaired Mann–Whitney test. Results are presented as the mean values ± SD (P value, ns-non-significant).

Supplementary Figure 6 Ndufs4(-/-) CD4+ T cells are characterized by abnormally elevated ROS production. **(A)** Representative flow cytometry stacked histogram plot of 2NBDG-FITC intensity gated on CD3- (non-CD3, control), WT, and Ndufs4(-/-) CD4+ T cells. **(B)** Bar graph summarizing the results of A, as MFI of 2NDBG-FITC. **(C)** Representative flow cytometry stacked histograms of MitoSox® staining gated on CD4+ T cells of naïve or activated splenocytes from WT and Ndufs4(-/-) mice. WT CD4+ T cells were used as controls, with the following conditions from top to bottom: unstained, pre-treated with 200µM N Acetyl Cysteine (NAC), and treated with rotenone and antimycin A (Rot/AA). **(D)** Bar graph summarizing the results shown in **(C)**, as MFI of MitoSox. **(E, F)** Bar graphs demonstrating the area under the curve (AUC) values of the omega-3 fatty acids Docosahexaenoic acid (DHA) and Eicosapentaenoic acid (EPA), respectively, within the media fraction. AUCs were obtained from the 13C6-glucose tracing metabolome of CD8+ T cells of WT and Ndufs4(-/-) littermate mice, as explained in Figure 10. Statistical method: two-tailed unpaired Mann–Whitney test. Results are presented as the mean values ± SD. (P value, ns-non significant; *P≤0.05, **P≤0.01 and ****P≤0.0001).

Supplementary Figure 7 CD4+ T-cell subsets, activation, and proliferation in a patient with NDUFS4 loss-of-function variant. **(A)** Complete peripheral blood count, including myeloid and lymphoid cell lines, of P1 vs. HC. **(B)** T-cell receptor (TCR) V-β repertoire of P1, as compared to HC. **(C)** Flow cytometry density plots of naïve, memory, central memory (TCM) and effector memory (TEM) CD4+ cells of P1, his heterozygous mother, and HC. **(D)** Summary of naïve, memory, TCM and TEM subsets in P1, his heterozygous parents, and HC. **(E)** Representative flow cytometry stacked histogram of CD25 intensity gated on CD4+ T cells from PBMCs that were activated as previously detailed in **Figure 5**. **(F)** Bar graph summarizing the results shown in F, as mean fluorescence intensity (MFI) of CD25 staining. **(G)** Representative stacked histogram of CellTrace violet intensity (CTV) of CD4+ T cells that were activated for 5 days with plate-bound CD3/CD28 antibodies. **(H)** Bar graph summarizing the results in G, as a proliferation index. **(I)** CD8+ T cells were stained with propidium iodide (PI) to measure activation-induced cell death (AICD). Presented is a density plot of P1, his heterozygous mother and a HC. **(J)** Bar graph summarizing the results of I. PI+ CD8+ T cells are presented as percentages. **(K, L)** Analysis of AICD in CD4+ T cells following activation, as mentioned above.

Supplementary Figure 8 Sequential, step-by-step gating approach for analyzing the flow-cytometry data presented in **Figure 1**. **(A–C)** Gating strategy for lymphocytes. Cells were first gated on forward scatter (FSC) versus side scatter (SSC) to exclude debris and identify the main lymphocyte population **(A)**. Singlets were then selected using an FSC-A versus FSC-H gate to remove doublets **(B)**. From the singlet population, cells were subsequently gated on CD4 and CD8 expression **(C)**. **(D–F)** Gating strategy for thymocytes. Panels **(D, E)** correspond to the same FSC/SSC and singlet gates described in A and B, respectively. From the singlet population, thymocytes were then gated on TCRβ and CD69 **(F)**. **(G–L)** Gating strategy for homeostatic expansion experiments. Panels G and H follow the same FSC/SSC **(G)** and singlet **(H)** gating steps as above. CD8^+^ cells were next identified using CD8 versus FSC A **(I)**. To define naïve T cells (TN), CD8^+^ cells were gated on CD44^-^CD62L^+^**(J)**. Finally, CellTrace signal intensity for TN cells from CD45.1+ and CD45.2+ mice is shown as a histogram **(K–L)**.

Supplementary Figure 9 Sequential, step-by-step gating approach for analyzing the flow-cytometry data presented in **Figures 2**, **3**. **(A–D)** Gating strategy for the in vivo infection model used to assess CD8^+^ T-cell activation. Cells were first gated on forward scatter (FSC) versus side scatter (SSC) to identify the main population **(A)**. Singlets were then selected using an SSC-H versus SSC-A plot to exclude doublets **(B)**. From the singlet gate, CD45^+^ leukocytes were identified **(C)** and subsequently gated on CD8^+^Vα2^+^ T cells **(D)**. **(F–J)** Gating strategy for the in vivo adoptive T-cell transfer experiments. Panels F and G correspond to the same FSC/SSC **(F)** and singlet **(G)** gates described in A and B. From the singlet population, CD45.2^+^ donor T cells were selected **(H)** and further gated on CD8^+^Vα2^+^ expression **(I)**. Finally, CellTrace fluorescence intensity for CD8^+^Vα2^+^ donor T cells (CD45.2^+^) is presented as a histogram **(J)**.

Supplementary Figure 10 Sequential, step-by-step gating approach for analyzing the flow-cytometry data presented in **Figure 5**. **(A–D)** Gating strategy for the 2-NDBG glucose uptake assay. Cells were first gated on forward scatter (FSC) versus side scatter (SSC) to identify the main population **(A)**. Singlets were then selected using an FSC-H versus FSC-A plot to exclude doublets **(B)**. From the singlet population, CD8^+^ T cells were identified **(C)**, and 2 NDBG fluorescence intensity for the gated CD8^+^ T cells is displayed as a histogram **(D)**. **(E–H)** Gating strategy for Mitosox-based reactive oxygen species (ROS) analysis in naïve T cells. Panel E corresponds to the FSC/SSC gate used in A. Singlets were identified using an SSC-H versus SSC-A plot **(F)**. Naïve T cells were then selected by gating on CD44^-^CD62L^+^ cells **(G)**. Finally, Mitosox Red fluorescence intensity for CD8^+^ CD44^-^CD62L^+^ naïve T cells is shown as a histogram **(H)**.

Supplementary Figure 11 Sequential, step-by-step gating approach for analyzing the flow-cytometry data presented in **Figure 6**. **(A-D)** Gating strategy for samples from a patient with Leigh syndrome. Cells were first gated on forward scatter (FSC) versus side scatter (SSC) to identify the main population **(A)**. Singlets were then selected using an FSC-A versus FSC-H gate to exclude doublets **(B)**. From the singlet population, CD8^+^ T cells were identified **(C)**, and effector versus central memory subsets were defined by gating on CD44 and CD62L expression **(D)**. **(E–J)** Gating strategy for T-cell activation and proliferation analyses in the patient. Panels **(E-G)** correspond to the FSC/SSC, singlet, and CD4/CD8 gates shown in A, B, and C, respectively. Activated T cells were then identified by gating CD8^+^ T cells on CD25 expression **(H)**. CellTrace fluorescence intensity for activated CD8^+^CD25^+^ T cells is shown as a histogram **(I)**. Additionally, T-cell activation was assessed by intracellular IFNγ staining, measured by gating on CD8 and IFNγ expression **(J)**.

Supplementary Table 1 Immune workup of the patient with NDUFS4 loss-of-function variant. NDUFS4- NADH: ubiquinone oxidoreductase iron-sulfur protein 4; TCM- central memory T cells; TEM – effector memory T cells; Tregs – Regulatory T cells; FOXP3- Forkhead box P3; NK- Natural killer; Ig- Immunoglobulins; HBV- Hepatitis B virus; EBV- Epstein-Barr virus; VCA Viral Capsid Antigen; EBNA- EBV nuclear antigen; VZV- Varicella Zoster Virus; CMV- Cytomegalovirus. In Bold and italics - values are above and below normal reference range, respectively. * Age-matched reference ranges for T cell subsets are taken from Garcia-Prat et al.37; Age-matched IgG, IgM and IgA reference ranges are taken from Jolliff et al 36.

Supplementary Table 2 List of primers used in PCR for mice genotyping. * Myogenin was used as DNA loading control together with the Lck-cre primers; ** Used for validation of NDUFS4 (-/-) following excision of exon 2 by the Lck-cre recombinase; *** Used as DNA loading control together with the OT-I primers.

Supplementary Table 3 Primers used for mitochondrial DNA quantification by sqRT-PCR. * Mito-RNR2 and β2 microglobulin (β2M) are representative genes of MtDNA and nDNA, respectively.
