# Supplementary material for: NDUFS4, a mitochondrial complex I subunit, is essential for T-cell metabolic fitness and immune function
Source: Front Immunol. 2026 Jan 7;16:1734203. doi: 10.3389/fimmu.2025.1734203 (PMC12819820; doi:10.3389/fimmu.2025.1734203)
Supplement: Supplementary Figure 1 — T-cell-specific Ndufs4(-/-) mice model. (A) Ndufs4(loxp/loxp) mice were cross–bred with distal (d)Lck-Cre+ mice. The Cre recombinase excised exon 2 in the Ndufs4 gene, specifically in T cells. (B) To confirm knockout of Ndufs4, polymerase chain reaction (PCR) using primers designed to amplify the Ndufs4 DNA following the excision of exon 2 by the Cre recombinase was utilized. Thus, enabling differentiation of Ndufs4(-/-) from other mice. (C) Naïve CD3+ T cells were separated from splenocytes of Ndufs4(-/-) and WT mice using an immunomagnetic negative selection-based cell isolation kit. CD3+ T cells were then lysed and transferred to a nitrocellulose membrane. Following blocking, an anti-Ndufs4 probe was used to detect the Ndufs4 protein. Anti-β-tubulin antibody was used in the immunoblotting as a loading control. (D) Representative PCR of Cre+ mice. Myogenin is used as a DNA loading control. (E) Representative PCR of Ndufs4(loxp/loxp) mice. [file DataSheet1.pdf]

Supplementary data for: NDUFS4, a Mitochondrial Complex I Subunit, is Essential for T-Cell Metabolic Fitness and Immune Function

Oded Shamriz, Zahala Bar-On, Omri Yosef, Leonor Cohen-Daniel, Ayelet Sheer, Or Reuven, Wajeeh Salaymeh, Amijai Saragovi, Raz Somech, Atar Lev, Hagar Mor-Shaked, Yuval Tal, Aviva Fattal-Valevski, Simon Edvardson, Michael Berger

Correspondence: michaelb@ekmd.huji.ac.il (M.B); oded.shamriz@mail.huji.ac.il (O.D);

This file contains:

Supplementary Figures 1-11

Supplementary Tables 1-3

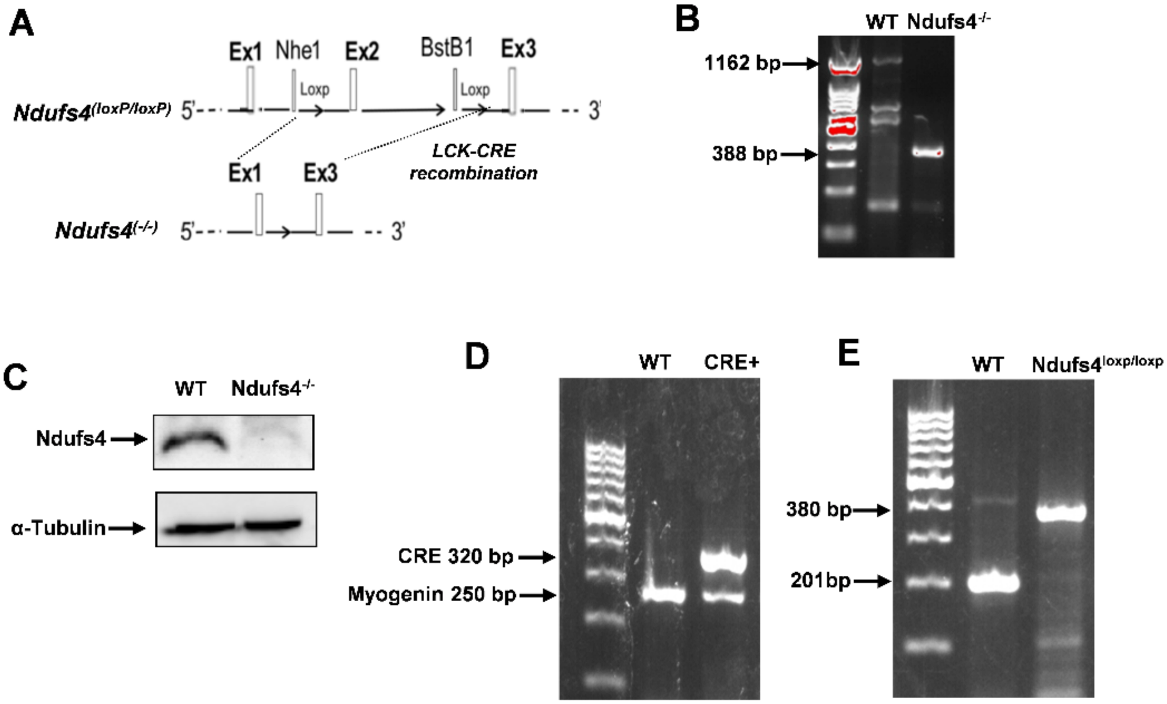

Figure S1: T-cell-specific *Ndufs4*$^{(-/-)}$ mice model. (A) *Ndufs*4$^{(loxp/loxp)}$ mice were cross–bred with distal (d)Lck-Cre^{+} mice. The Cre recombinase excised exon 2 in the *Ndufs4* gene, specifically in T cells. (B) To confirm knockout of *Ndufs4*, polymerase chain reaction (PCR) using primers designed to amplify the *Ndufs4* DNA following the excision of exon 2 by the Cre recombinase was utilized. Thus, enabling differentiation of *Ndufs4*$^{(-/-)}$ from other mice. (C) Naïve CD3^{+} T cells were separated from splenocytes of *Ndufs4*$^{(-/-)}$ and WT mice using an immunomagnetic negative selection-based cell isolation kit. CD3^{+} T cells were then lysed and transferred to a nitrocellulose membrane. Following blocking, an anti-*Ndufs4* probe was used to detect the *Ndufs4* protein. Anti-β-tubulin antibody was used in the immunoblotting as a loading control. (D) Representative PCR of Cre^{+} mice. *Myogenin* is used as a DNA loading control. (E) Representative PCR of *Ndufs4*$^{(loxp/loxp)}$ mice.

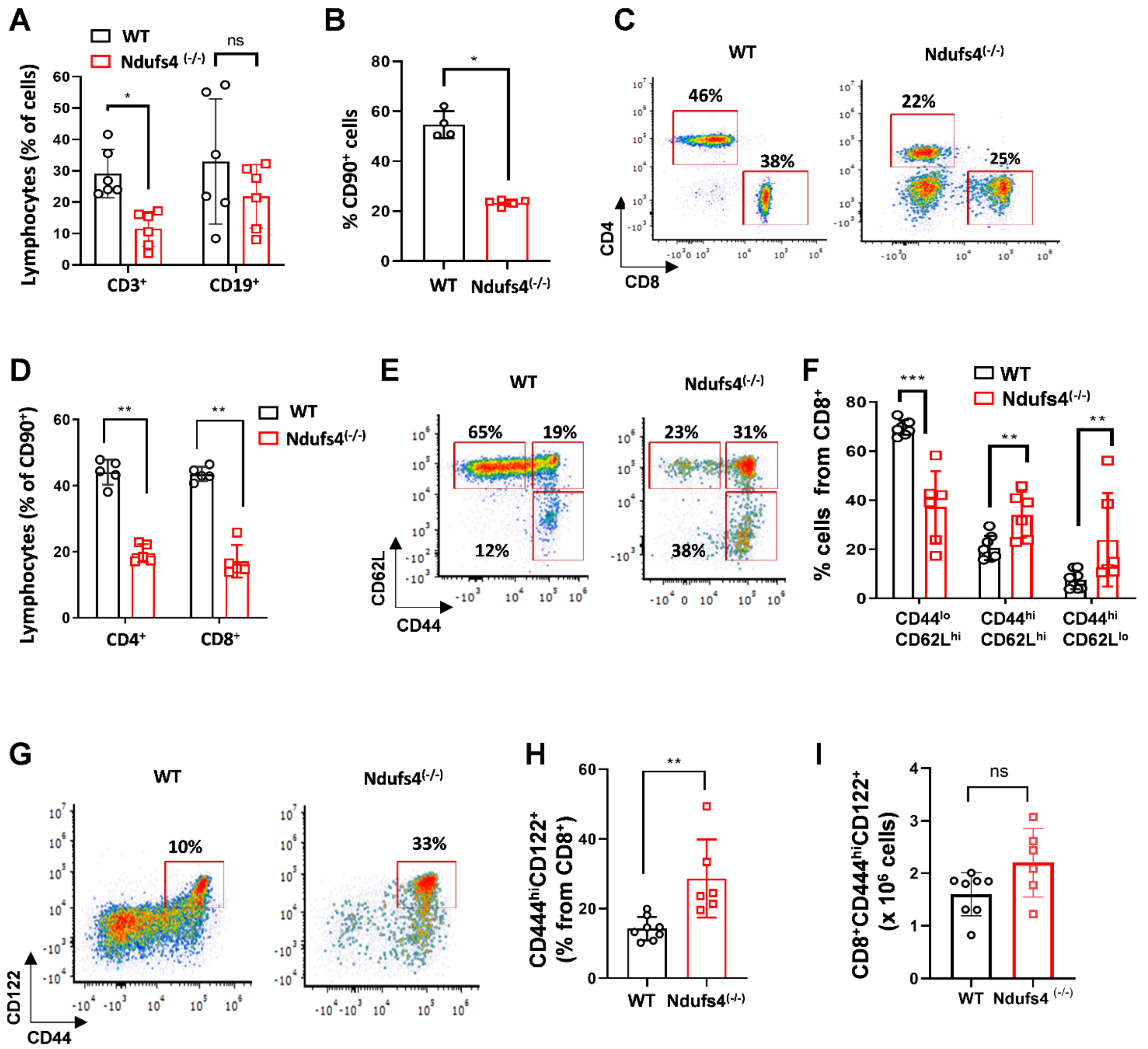

Figure S2: Immune phenotyping of peripheral blood, lymph nodes, and spleens of *Ndufs4*$^{(-/-)}$ mice. (A) Peripheral blood mononuclear cells (PBMC) of *Ndufs4*$^{(-/-)}$ and WT mice were stained for CD19 and CD3. Bar graph summarizing the results (n=6 mice, for each group). (B) *Ndufs4*$^{(-/-)}$ and WT mice were sacrificed, and cervical lymph nodes were extracted and stained for lymphocyte markers. Bar graph summarizing results of flow-cytometry analysis of CD90, a pan-T cell marker (n=4 and 6 mice for WT and *Ndufs4*$^{(-/-)}$mice, respectively). (C) Representative flow-cytometry of CD4^{+} and CD8^{+} T cells derived from cervical lymph nodes of *Ndufs4*$^{(-/-)}$ and WT mice. (D) Bar graph summarizing results of C (n=6 mice, for each group). (E) Representative flow cytometry plots showing the frequencies of naïve (CD44^{lo}CD62L^{hi}), T$_{CM}$ (CD44^{hi}CD62L^{hi}), and T$_{EM}$ (CD44^{hi}CD62L^{lo}) subsets of CD8^{+} T cells in the spleens derived from WT and *Ndufs4*$^{(-/-)}$ littermate mice. (F) Bar graph summarizing the results of E, indicating percentages (n=6 mice, for each group). (I) Same as H, summarizing absolute cell numbers of CD8^{+}CD44^{hi}CD122^{hi} T cells. (n=6 mice, for each group). (G, H) Same as E and F, respectively, for the CD44^{lo}CD122^{hi} subset of CD8^{+} T cells. (n=6 mice, for each group). Statistical method: two-tailed unpaired Mann–Whitney test. Results are presented as the mean values ± SD (P value, *ns-non-significant*; *P≤0.05, **P≤0.01, *** P≤0.001).

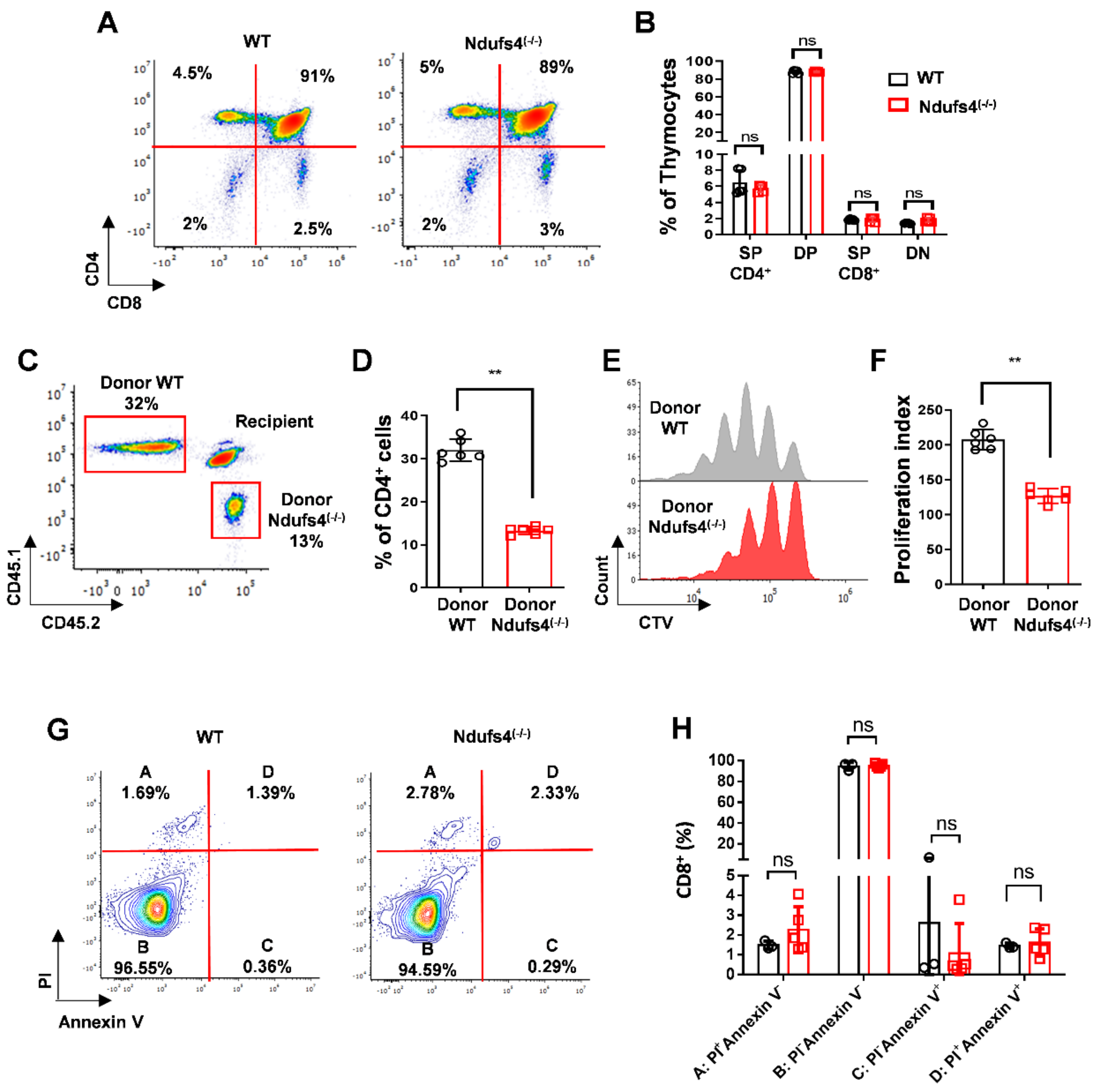

Figure S3: Intact thymic subsets and impaired homeostatic expansion of $CD4^+$ T cells is seen in *Ndufs4*$^{(-/-)}$ mice. (A) Representative flow cytometry plots demonstrating the frequencies of single positive ($CD4^+$ or $CD8^+$), double positive ($CD4^+CD8^+$), and double negative ($CD4^-CD8^-$) thymocytes derived from 5-week-old *Ndufs4*$^{(-/-)}$ and WT littermate mice. (B) Bar graph summarizing the results of A (n=5 mice, for each group). (C) Representative flow cytometry plots of CD45.1 vs. CD45.2 staining of $CD4^+$ splenocytes from recipient mice, 7 days after cell transfer. The Homeostatic expansion experiment was previously detailed in *Figure 6*. (D) Bar graph summarizing the results shown in C. (E) Representative flow cytometry stacked histogram of Cell-Trace violet (CTV) intensity of donor $CD4^+$ T cells. (F) Bar graph summarizing the results shown in E, as proliferation index (n=6 mice, for each group). (G) Representative flow cytometry plots demonstrating the frequencies of Propidium iodide $(PI)^+$ Annexin V^- (A), PI^-Annexin V^- (B), PI^- Annexin V^+ (C), and PI^+ Annexin V^+ (D) $CD8^+$ splenocytes from WT and *Ndufs4* $^{(-/-)}$ mice. (H) Bar graph summarizing the results of A. Statistical method: two-tailed unpaired Mann–Whitney test. Results are presented as the mean values ± SD (*P value*, ns- non-significant*, **P≤0.01*).

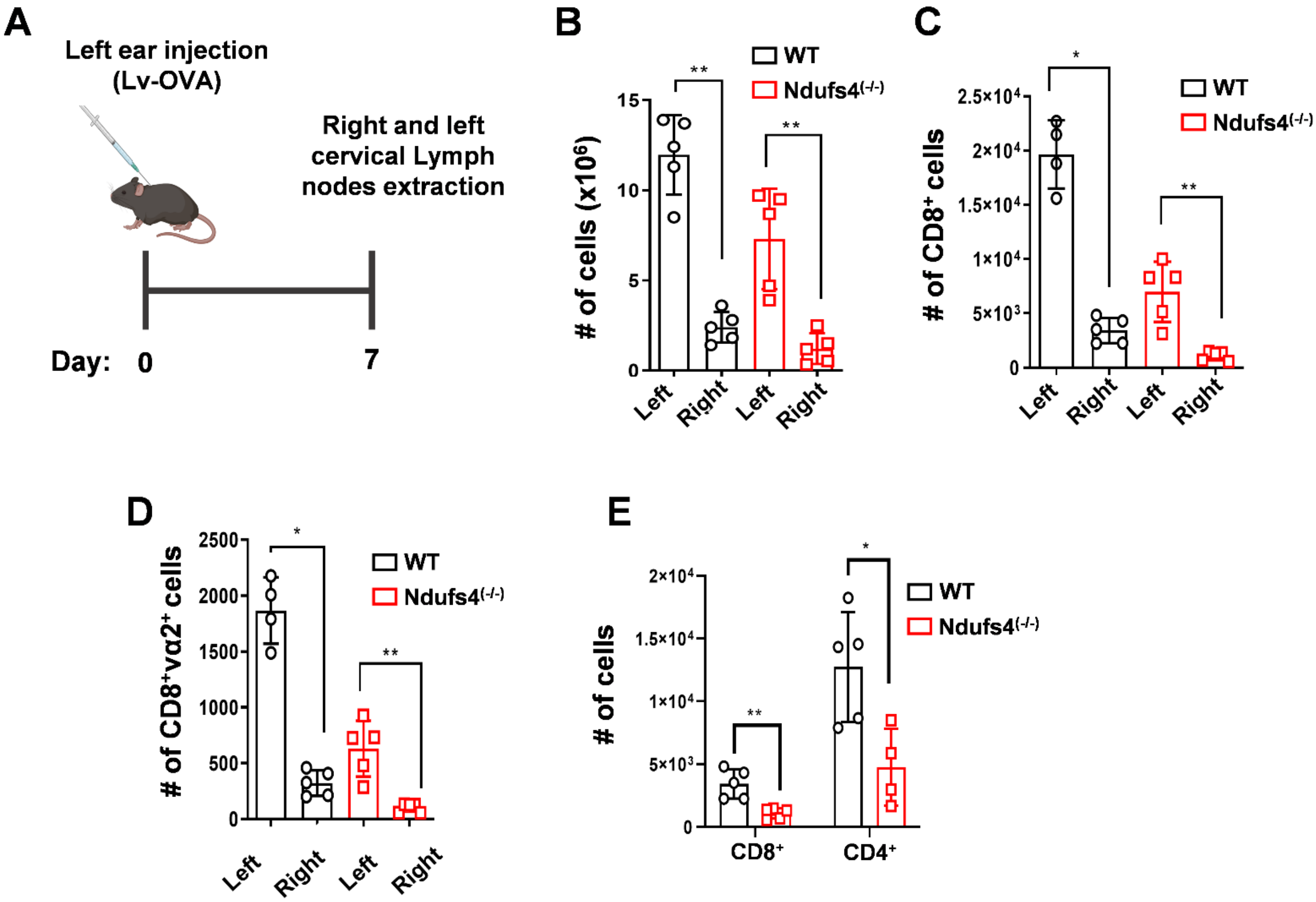

Figure S4: Controls for the *in-vivo* infection model used to study CD8^{+} T cell activation. (A) In-vivo model examining CD8^{+} T-cell effector functions. Lv-OVA was injected to the left ear pinnae of *Ndufs4*$^{(-/-)}$ and WT littermate mice. Seven days later, left cervical lymph nodes were extracted and analyzed by flow cytometry; right cervical lymph nodes were used as negative controls, as right ear pinnae of each mouse were not exposed to the Lv-OVA. (B) Bar graph comparing cell counts of right and left cervical lymph nodes in *Ndufs4*$^{(-/-)}$ and WT littermate mice (n=5 mice, for each group). (C, D) Same as B, with counts of CD8^{+} and CD8^{+}Vα2^{+} T cells, respectively (n=5 mice, for each group). (E) Bar graph summarizing cell counts of CD4^{+} and CD8^{+} T cells derived from right cervical lymph nodes of each *Ndufs4*$^{(-/-)}$ and WT littermate mice (n=5 mice, for each group). Statistical method: two-tailed unpaired Mann–Whitney test. Results are presented as the mean values ± SD (P value, ns-non-significant; *P≤0.05, **P≤0.01).

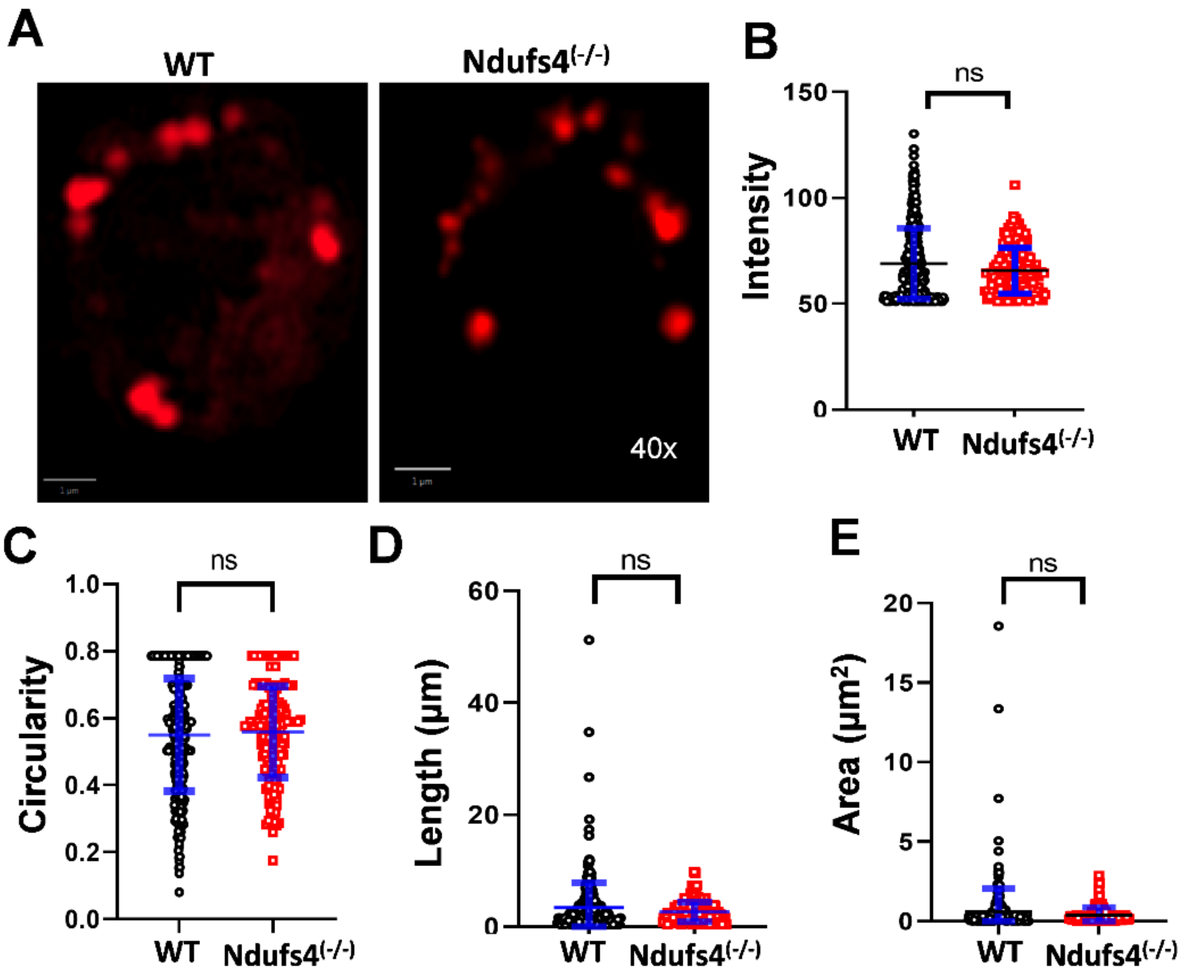

Figure S5: Confocal microscopy of mitochondria in naïve *Ndufs4* $^{(-/-)}$ T cells. (A) *Ndufs4* $^{(-/-)}$ and WT littermate mice were sacrificed, and their spleens were harvested. $CD8^+$ T cells were separated using an immunomagnetic negative selection kit, and a mixture was prepared for each mouse group. Mitochondria were then stained with MitoTracker® far-red and visualized by confocal microscopy (n=3 mice, for each group). (B-E) Dot plots summarizing mitochondrial intensity, circularity, length, and surface area, respectively, of $CD8^+$ T cells derived from *Ndufs4* and WT littermate mice. Each dot in the graphs represents a single mitochondrion. Statistical method: two-tailed unpaired Mann–Whitney test. Results are presented as the mean values ± SD (P value, ns-non-significant).

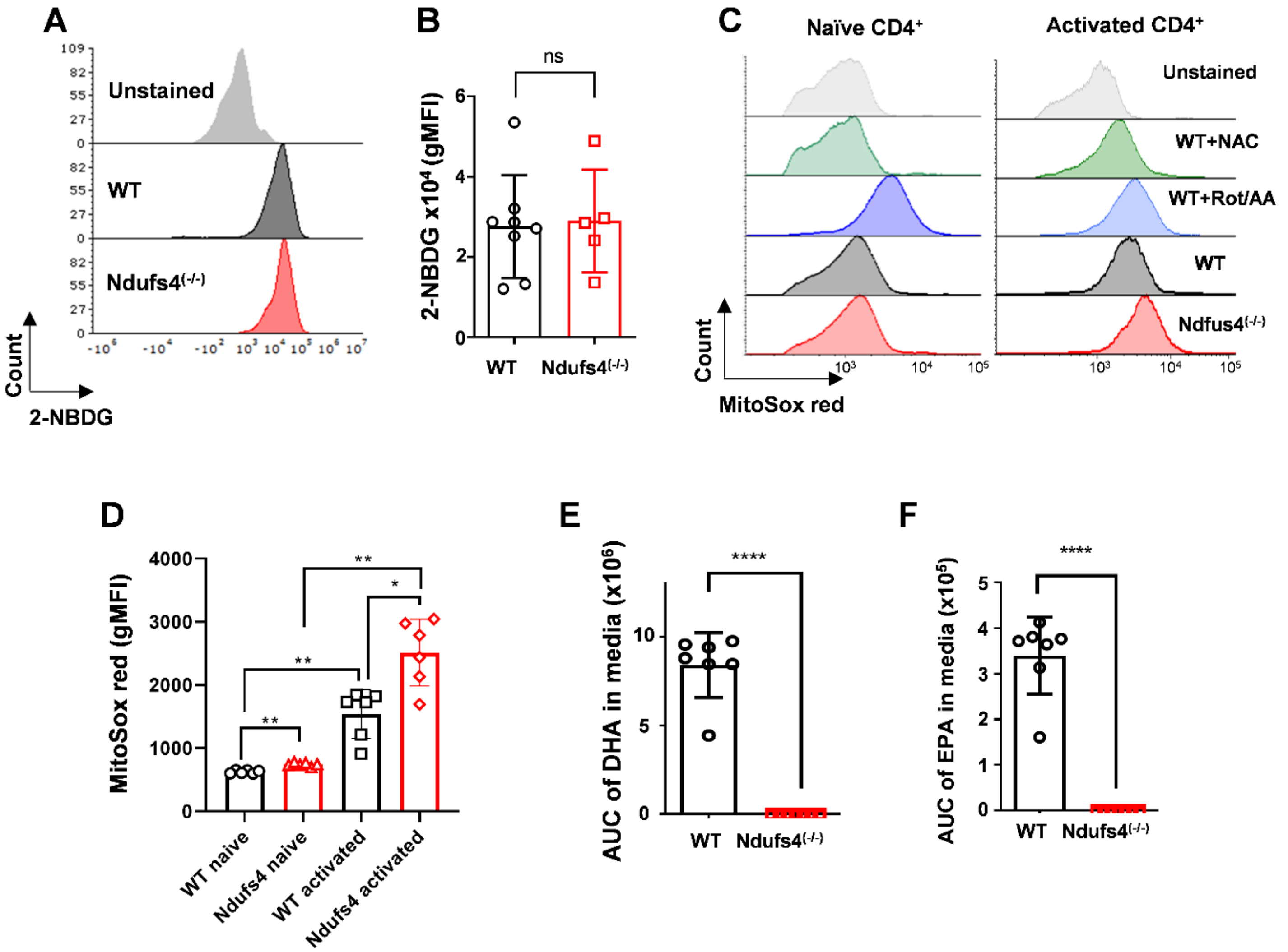

Figure S6: *Ndufs4*$^{(-/-)}$ CD4^{+} T cells are characterized by abnormally elevated ROS production. (A) Representative flow cytometry stacked histogram plot of 2NBDG-FITC intensity gated on CD3^{-} (non-CD3, control), WT, and *Ndufs4*$^{(-/-)}$ CD4^{+} T cells. (B) Bar graph summarizing the results of A, as MFI of 2NDBG-FITC. (C) Representative flow cytometry stacked histograms of MitoSox® staining gated on CD4^{+} T cells of naïve or activated splenocytes from WT and *Ndufs4*$^{(-/-)}$ mice. WT CD4^{+} T cells were used as controls, with the following conditions from top to bottom: unstained, pre-treated with 200µM N-Acetyl Cysteine (NAC), and treated with rotenone and antimycin A (Rot/AA). (D) Bar graph summarizing the results shown in C, as MFI of MitoSox. (E, F) Bar graphs demonstrating the area under the curve (AUC) values of the omega-3 fatty acids Docosahexaenoic acid (*DHA)* and Eicosapentaenoic acid (EPA), respectively, within the media fraction. AUCs were obtained from the $^{13}C_6$-glucose tracing metabolome of CD8^{+} T cells of WT and *Ndufs4*$^{(-/-)}$ littermate mice**,** as explained in *Figure 10.* Statistical method: two-tailed unpaired Mann–Whitney test. Results are presented as the mean values ± SD. (*P value*, *ns-non-significant; *P≤0.05, **P≤0.01 and ****P≤0.0001)*

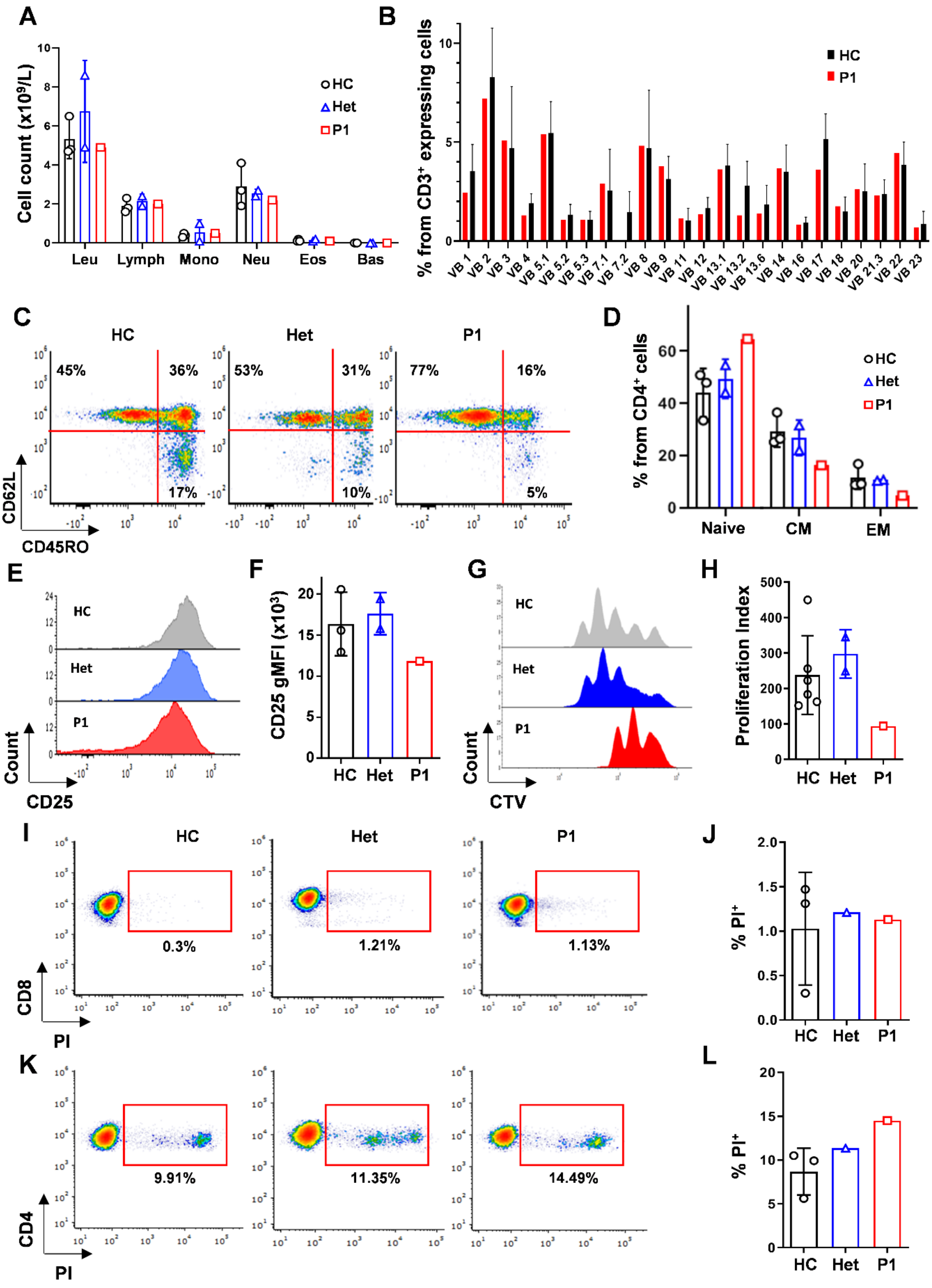
A
Cell count (x10⁹/L)
HC
Het
P1
Leu
Lymph
Mono
Neu
Eos
Bas
B
% from CD3+ expressing cells
HC
P1
VB 1
VB 2
VB 3
VB 4
VB 5.1
VB 5.2
VB 5.3
VB 7.1
VB 7.2
VB 8
VB 9
VB 11
VB 12
VB 13.1
VB 13.2
VB 13.6
VB 14
VB 16
VB 17
VB 18
VB 20
VB 21.3
VB 22
VB 23
C
HC
Het
P1
45%
36%
17%
53%
31%
10%
77%
16%
5%
CD62L
CD45RO
D
% from CD4+ cells
HC
Het
P1
Naive
CM
EM
E
HC
Het
P1
Count
CD25
F
CD25 gMFI (x10³)
HC
Het
P1
G
HC
Het
P1
Count
CTV
H
Proliferation Index
HC
Het
P1
I
HC
Het
P1
0.3%
1.21%
1.13%
CD8
PI
J
% PI+
HC
Het
P1
K
9.91%
11.35%
14.49%
CD4
PI
L
% PI+
HC
Het
P1

Figure S7: CD4$^+$ T-cell subsets, activation, and proliferation in a patient with NDUFS4 loss-of-function variant. (A) Complete peripheral blood count, including myeloid and lymphoid cell lines, of P1 vs. HC. (B) T-cell receptor (TCR) V-β repertoire of P1, as compared to HC. (C) Flow cytometry density plots of naïve, memory, central memory (T_{CM}) and effector memory (T_{EM}) CD4$^+$ cells of P1, his heterozygous mother, and HC. (D) Summary of naïve, memory, T_{CM} and T_{EM} subsets in P1, his heterozygous parents, and HC. (E) Representative flow cytometry stacked histogram of CD25 intensity gated on CD4$^+$ T cells from PBMCs that were activated as previously detailed in *Figure 5.* (F) Bar graph summarizing the results shown in F, as mean fluorescence intensity (MFI) of CD25 staining. (G) Representative stacked histogram of CellTrace violet intensity (CTV) of CD4$^+$ T cells that were activated for 5 days with plate-bound CD3/CD28 antibodies. (H) Bar graph summarizing the results in G, as a proliferation index. (I) CD8+ T cells were stained with propidium iodide (PI) to measure activation-induced cell death (AICD). Presented is a density plot of P1, his heterozygous mother and a HC. (J) Bar graph summarizing the results of I. PI$^+$ CD8$^+$ T cells are presented as percentages. (K, L) Analysis of AICD in CD4$^+$ T cells following activation, as mentioned above.

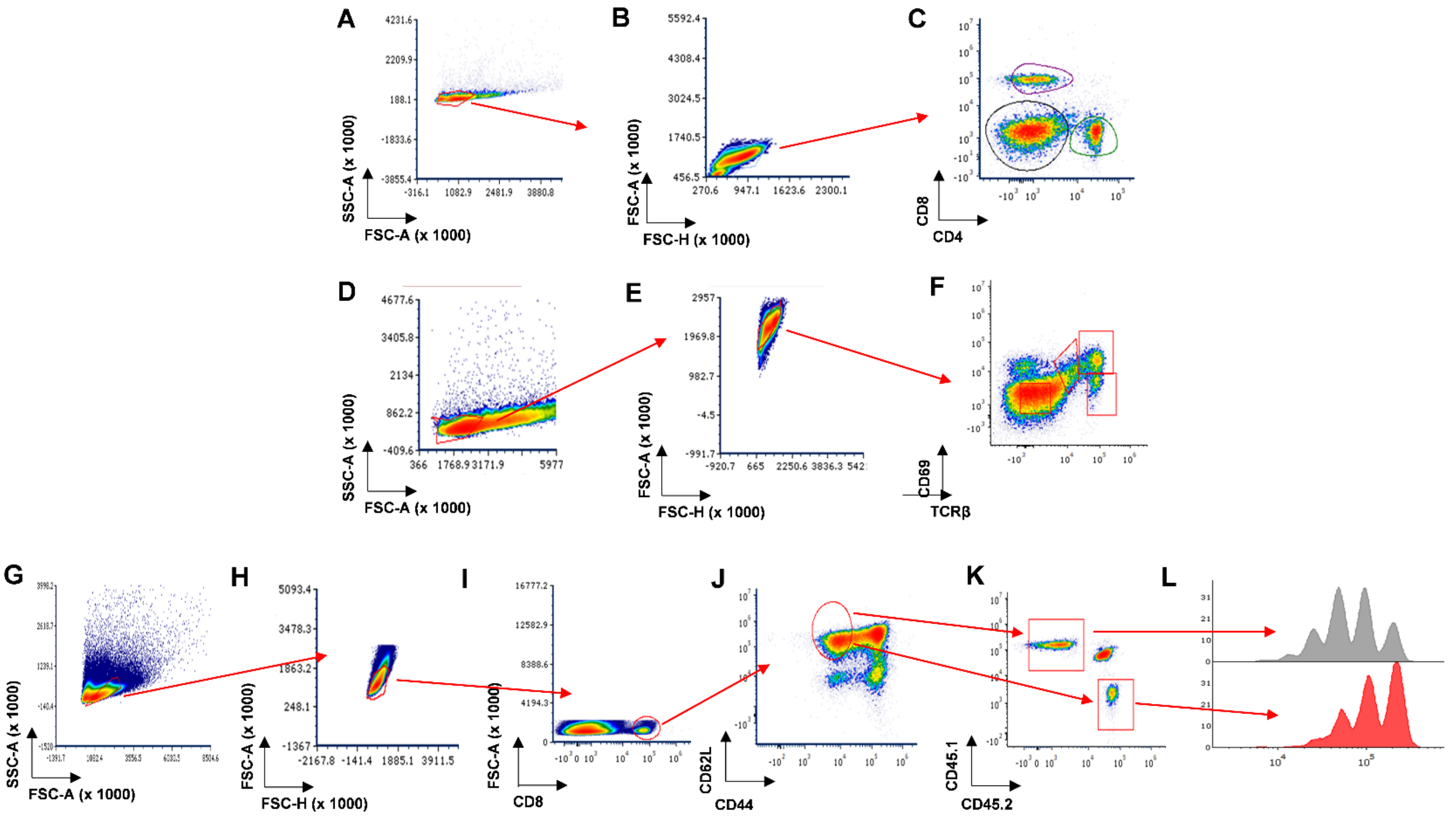

Figure S8: Sequential, step-by-step gating approach for analyzing the flow-cytometry data presented in Fig. 1. (A–C) *Gating strategy for lymphocytes.* Cells were first gated on forward scatter (FSC) versus side scatter (SSC) to exclude debris and identify the main lymphocyte population (A). Singlets were then selected using an FSC-A versus FSC-H gate to remove doublets (B). From the singlet population, cells were subsequently gated on CD4 and CD8 expression (C). (D–F) *Gating strategy for thymocytes.* Panels D and E correspond to the same FSC/SSC and singlet gates described in A and B, respectively. From the singlet population, thymocytes were then gated on TCRβ and CD69 (F). (G–L) *Gating strategy for homeostatic expansion experiments.* Panels G and H follow the same FSC/SSC (G) and singlet (H) gating steps as above. CD8$^+$ cells were next identified using CD8 versus FSC-A (I). To define naïve T cells (T_N), CD8$^+$ cells were gated on CD44$^-$CD62L$^+$ (J). Finally, CellTrace signal intensity for T_N cells from CD45.1$^+$ and CD45.2$^+$ mice is shown as a histogram (K–L).

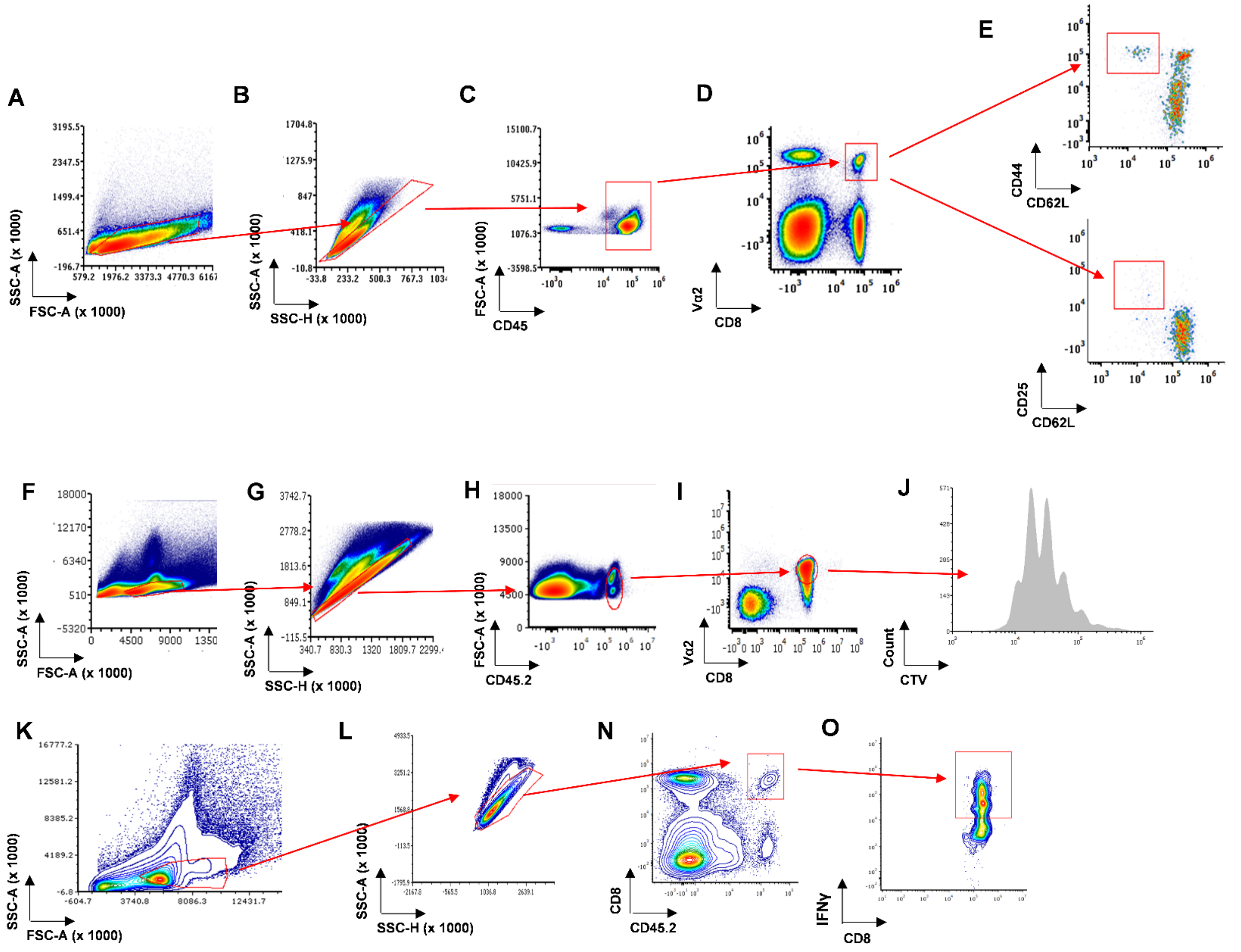

Figure S9: Sequential, step-by-step gating approach for analyzing the flow-cytometry data presented in Fig. 2 and Fig. 3. (A–D) *Gating strategy for the in vivo infection model used to assess CD8$^+$ T-cell activation.* Cells were first gated on forward scatter (FSC) versus side scatter (SSC) to identify the main population (A). Singlets were then selected using an SSC-H versus SSC-A plot to exclude doublets (B). From the singlet gate, CD45$^+$ leukocytes were identified (C) and subsequently gated on CD8$^+$Vα2$^+$ T cells (D). (F–J) *Gating strategy for the in vivo adoptive T-cell transfer experiments.* Panels F and G correspond to the same FSC/SSC (F) and singlet (G) gates described in A and B. From the singlet population, CD45.2$^+$ donor T cells were selected (H) and further gated on CD8$^+$Vα2$^+$ expression (I). Finally, CellTrace fluorescence intensity for CD8$^+$Vα2$^+$ donor T cells (CD45.2$^+$) is presented as a histogram (J).

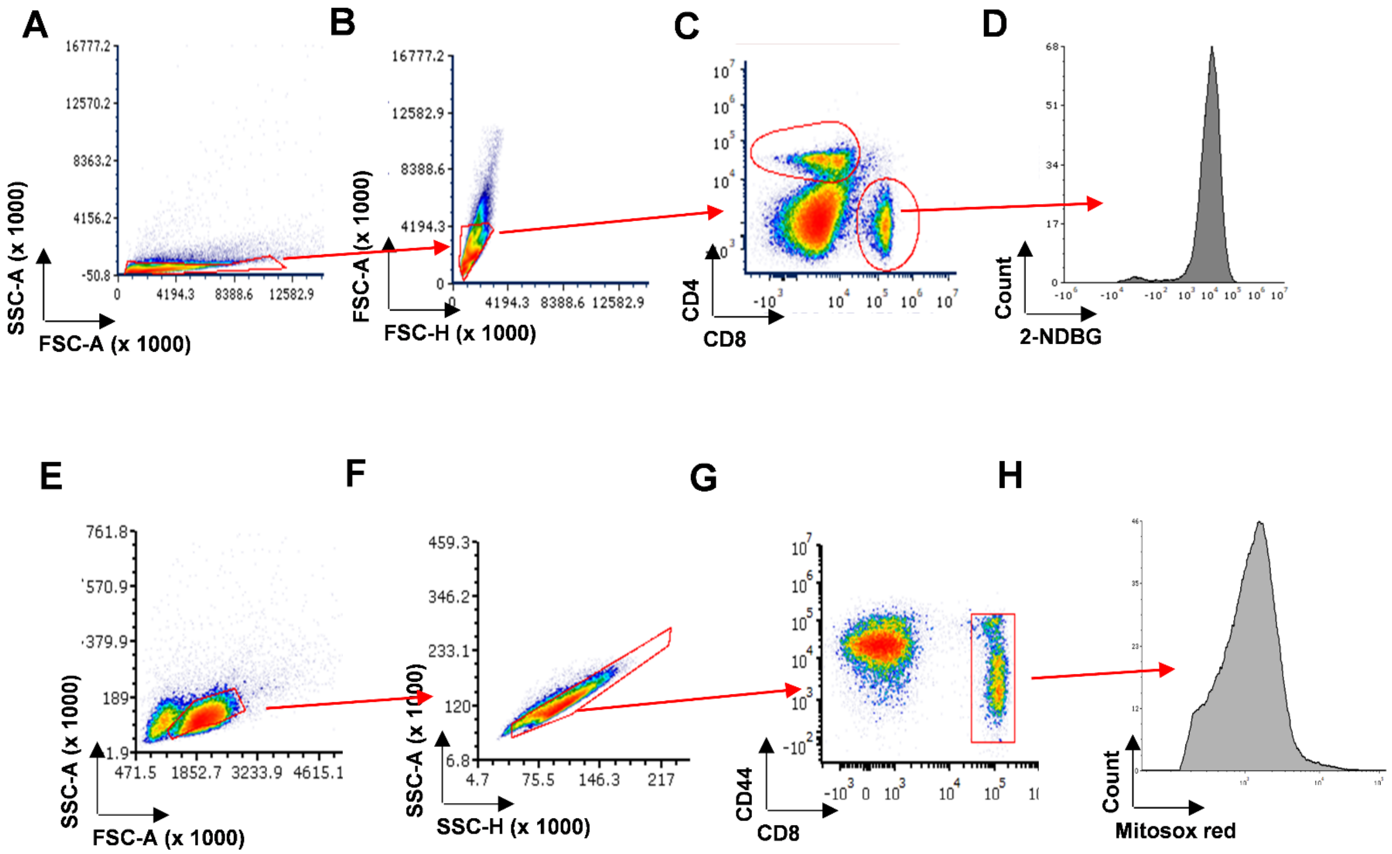

Figure S10: Sequential, step-by-step gating approach for analyzing the flow-cytometry data presented in Fig. 5. (A–D) *Gating strategy for the 2-NDBG glucose uptake assay.* Cells were first gated on forward scatter (FSC) versus side scatter (SSC) to identify the main population (A). Singlets were then selected using an FSC-H versus FSC-A plot to exclude doublets (B). From the singlet population, $CD8^+$ T cells were identified (C), and 2-NDBG fluorescence intensity for the gated $CD8^+$ T cells is displayed as a histogram (D). **(E–H)** *Gating strategy for Mitosox-based reactive oxygen species (ROS) analysis in naïve T cells.* Panel E corresponds to the FSC/SSC gate used in A. Singlets were identified using an SSC-H versus SSC-A plot (F). Naïve T cells were then selected by gating on $CD44^-CD62L^+$ cells (G). Finally, Mitosox Red fluorescence intensity for $CD8^+$ $CD44^-CD62L^+$ naïve T cells is shown as a histogram (H).

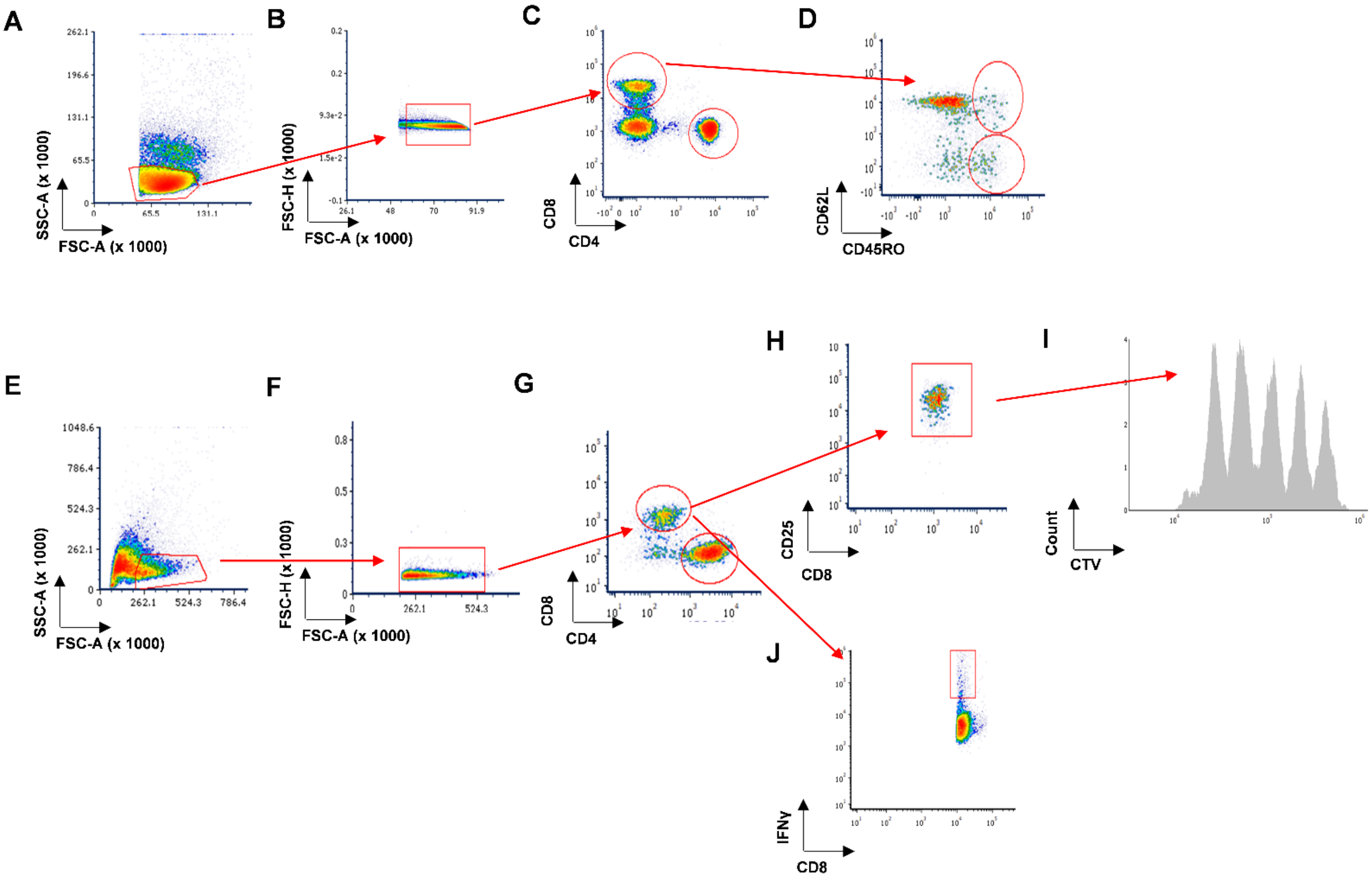

Figure S11: Sequential, step-by-step gating approach for analyzing the flow-cytometry data presented in Fig. 6. (A–D) *Gating strategy for samples from a patient with Leigh syndrome.* Cells were first gated on forward scatter (FSC) versus side scatter (SSC) to identify the main population (A). Singlets were then selected using an FSC-A versus FSC-H gate to exclude doublets (B). From the singlet population, $CD8^{+}$ T cells were identified (C), and effector versus central memory subsets were defined by gating on CD44 and CD62L expression (D). (E–J) *Gating strategy for T-cell activation and proliferation analyses in the patient.* Panels E, F, and G correspond to the FSC/SSC, singlet, and CD4/CD8 gates shown in A, B, and C, respectively. Activated T cells were then identified by gating $CD8^{+}$ T cells on CD25 expression (H). CellTrace fluorescence intensity for activated $CD8^{+}CD25^{+}$ T cells is shown as a histogram (I). Additionally, T-cell activation was assessed by intracellular IFNγ staining, measured by gating on CD8 and IFNγ expression (J).

Parameter				P1 (8 years)	Age-adjusted normal range *
Absolute leukocyte count (10^9/L)				4.9	4.50-13.50
Absolute lymphocyte count (10^9/L)				2	1.50-6.80
$CD3^+$ (%)				82.28	61.00-84.00
$CD4^+$ (%)	Total			40.32	26.00-53.00
	Naïve		$CD45RA^+CD45RO^-$	64.74	32.00-68.00
	Memory	T_{CM}	$CD45RO^+CD62L^+$	16.27	10.27-42.6
		T_{EM}	$CD45RO^+CD62L^-$	*4.68*	30.3-72.0
	T_{regs}		$CD4^+CD25^+FOXP3^+$	2.09	1.00-3.70
$CD8^+$ (%)	Total			20.23	19.00-35.00
	Naive		$CD54RA^+CD45RO^-$	**70.46**	30.00-61.00
	Memory	T_{CM}	$CD45RO^+CD62L^+$	4.19	0.0-27.6
		T_{EM}	$CD45RO^+CD62L^-$	9.12	7.6-74.1
NK cells (%)	$CD56^+$			10.42	8.00-22.00
B cells (%)	$CD19^+$			*7.25*	8.00-23.00
Serum Ig			IgG (mg/dL)	860	633.00-1280.00
			IgA (mg/dL)	274	33.00-202.00
			IgM (mg/dL)	51.3	48.00-207.00
			IgE (IU/mL)	4.56	1.03-161.3
Specific IgG antibodies (U/mL)			Rubella	38.1	30.00<
			VZV	1116	135.00<
			Mumps	99.9	8.90<
			Measles	85.1	13.50<
			CMV	*<5.00*	6.0<
			EBV VCA	338	20.0<
			EBV EBNA	>600	20.0<

Table S1: Immune workup of the patient with *NDUFS4* loss-of-function variant. *NDUFS4*- NADH: ubiquinone oxidoreductase iron-sulfur protein 4; T_{CM}- central memory T cells; T_{EM} – effector memory T cells; T_{regs} – Regulatory T cells; FOXP3- Forkhead box P3; NK- Natural killer; Ig- Immunoglobulins; HBV- Hepatitis B virus; EBV- Epstein-Barr virus; VCA- Viral Capsid Antigen; EBNA- EBV nuclear antigen; VZV- Varicella Zoster Virus; CMV- Cytomegalovirus. In Bold and *italics* - values are above and below normal reference range, respectively. * Age-matched reference ranges for T cell subsets are taken from Garcia-Prat et al.[37]; Age-matched IgG, IgM and IgA reference ranges are taken from Jolliff et al [36].

Gene	Forward primer	Reverse primer
Ndufs4 loxp/loxp	**5'** AGT-CAG-CAA-CAT-TTT-GGC-AGT **3'**	**5'** GAG-CTT-GCC-TAG-GAG-GAG-GT **3'**
Lck-cre +	**5'** GAA-CCT-GAT-GGA-CAT-GTT-CAG-G **3'**	**5'** AGT-GCG-TTC-GAA-CGC-TAG-GGC-CTG **3'**
Myogenin + *	**5'** TTA-CGT-CCA-TCG-TGG-ACA-GC-**3'**	**5'** TGG-GCT-GGG-TST-TAG-CCT-TA **3'**
Ndufs4 (-/-) **	**5'** GGA-GAG-ACA-AGG-AGCCTG-TT **3'**	**5'** GTG-GAG-AGCTTG-CCT-AGG-AG **3'**
OT-I	**5'** CAG CAG CAG GTG AGA CAA AGT **3'**	**5'** GGC TTT ATA ATT AGC TTG GTC C **3'**
OT-I internal control ***	**5'** CAA ATG TTG CTT GTC TGG TG **3'**	**5'** GTC AGT CGA GTG CAC AGT TT **3'**

Table S2: List of primers used in PCR for mice genotyping. * Myogenin was used as DNA loading control together with the Lck-cre primers; ** Used for validation of NDUFS4 (-/-) following excision of exon 2 by the Lck-cre recombinase; *** Used as DNA loading control together with the OT-I primers.

Gene*	Forward primer	Reverse primer
Mito-RNR2	**5'-** CTA-GAA-ACC-CCG-AAA-CCA-AA-**3'**	**5'-** CCA-GCT-ATC-ACC-AAG-CTC-GT-**3'**
β2M	**5'-** TGAG-GCT-TAT-TGC-AAT-GCT-G-**3'**	**5'-** ATG-GCG-GTT-ACA-GTC-CAA-AG-**3'**

Table S3: Primers used for mitochondrial DNA quantification by sqRT-PCR. * Mito-RNR2 and β2 microglobulin (β2M) are representative genes of MtDNA and nDNA, respectively.
